# Decoding of amidated aromatic C-terminus and sulfation by cholecystokinin receptors reveals conserved and divergent evolutionary mechanisms

**DOI:** 10.1016/j.jbc.2026.113327

**Published:** 2026-07-13

**Authors:** Jian-Hui Chang, Shao-Qian Wu, Xue-Ying Ding, Xing Pan, Hui-Ying Wang, Yu-Xuan He, Lin Xi, Yu-Shuo Mei, Cheng-Yi Liu, Cui-Ping Liu, Rui-Ting Mao, Fan Li, Yi-Long Zhang, Jiang-Yue Li, Guo Zhang, Jian Jing

**Affiliations:** 1 State Key Laboratory of Pharmaceutical Biotechnology, Department of Neurology and Medical Psychology, Nanjing Drum Tower Hospital, The affiliated Hospital of Nanjing University Medical School, Institute for Brain Sciences, School of Life Sciences, Nanjing University, Nanjing, Jiangsu, China; 2 Department of Pathophysiology, School of Basic Medical Sciences, Institute of Metabolism and Health, Henan University, Kaifeng, Henan, China

**Keywords:** cholecystokinin receptor, evolution, ligand recognition, receptor subtype selectivity, RFamide, tyrosine sulfation

## Abstract

Understanding how evolutionarily-related receptors preserve recognition principles for conserved peptide post-translational modifications (PTMs) while acquiring new selectivity remains central to neuropeptide-G protein-coupled receptor (GPCR) biology. The *Aplysia* cholecystokinin (CCK) system provides an informative model, as its peptides combine two representative PTM-related features: an amidated aromatic C-terminus (RFamide/DFamide), and a dual-tyrosine sulfation pattern exceeding the single-site architecture typical of vertebrates. Notably, its two receptor subtypes, apCCKR1 and apCCKR2, exhibit distinct sensitivities to ligands carrying different numbers of sulfated tyrosines, yet the molecular basis remains unclear. Here, we first used multiple docking approaches and found that CCK ligands could insert into receptor binding pockets in either N-terminal-in or C-terminal-in orientations. We then applied pose-specific mutagenesis to resolve this ambiguity and established a common C-terminal-in binding mode for apCCK peptides, similar to mammalian CCK/gastrin binding modes observed by cryo-EM. Within this framework, recognition of the C-terminal aromatic motif is hierarchically organized through three receptor positions, comprising a conserved anchor, a CCK-biased layer and a flexible tuning site. Moreover, we uncovered a mechanism for sulfation-number-dependent subtype selectivity: apCCKR1 accommodates an additional sulfate with minimal structural rearrangement, whereas apCCKR2 loses a favorable electrostatic network, disfavoring the disulfated ligand. Comparative analyses across representative lineages identified a conserved sulfate-recognition module alongside a lineage-restricted modifier, enabling inferences regarding the ancestral state of sulfation recognition and how it was differentially remodeled across protostomes and deuterostomes. Together, these findings establish a framework for understanding how homologous receptors may decode peptide chemical features through conserved structural logic and adaptive diversification.

G protein-coupled receptors (GPCRs) constitute the largest family of membrane receptors (1, 2, 3) and represent a major class of therapeutic targets (4, 5, 6, 7, 8). In neuropeptide signaling systems, understanding how receptors recognize conserved chemical features of peptide ligands is essential not only for explaining receptor activation and subtype selectivity, but also for elucidating how peptide-receptor systems diversify over evolution. Many neuropeptides retain deeply conserved chemical signatures, including post-translational modifications (PTMs) and characteristic sequence motifs (9, 10, 11, 12), yet homologous receptors do not necessarily interpret these features in the same manner. Thus, a fundamental question in neuropeptide-GPCR biology is to determine which aspects of ligand recognition are evolutionarily conserved, and which have been rewritten in a lineage- or subtype-specific context. Recent advances in high-resolution structural biology have significantly improved our understanding of neuropeptide–GPCR interactions (13, 14, 15, 16, 17, 18, 19, 20, 21, 22); however, experimentally determined structures remain limited, particularly for systems involving flexible ligands and multiple receptor conformational states (23, 24, 25). As a result, *in silico* modeling has become an increasingly important route to mechanistic interpretation (26, 27, 28, 29, 30, 31).

The cholecystokinin (CCK) signaling systems present across protostomes and deuterostomes provide a useful framework to address these questions. CCK-family peptides integrate two evolutionarily significant features: an amidated aromatic C-terminus (RFamide/DFamide) and sulfated tyrosines that further diversify ligand activity (32, 33, 34, 35, 36, 37, 38, 39, 40, 41, 42, 43, 44, 45). In general, for the C-terminus, CCKs in protostomes (including arthropods and lophotrochozoans) end in RFamide, but CCKs/gastrins in most deuterostomes end in DFamide (33, 37, 45). Currently, receptor-side decoding of the RF/DFamide-like motif has been investigated to some extent in cryo-EM studies of human receptors (17, 41, 42, 43, 44, 46), but have not been examined systematically in an evolutionary context, particularly in protostomes. Furthermore, in deuterostomes, sulfation of tyrosine has been shown to contribute to receptor-subtype selectivity, with CCK_A_R strongly preferring sulfated ligands, whereas the CCK_B_R discriminates much more weakly, an activation pattern of receptor subtypes observed in both birds and humans (41, 47). While structural studies in humans have begun to elucidate aspects of the mechanisms underlying this subtype selectivity (41, 42, 43), whether and how this recognition mode is maintained, remodeled, or diversified across protostomes and deuterostomes remains poorly understood.

Our previous work identified two endogenous CCK peptides in mollusk *Aplysia californica*, each containing two tyrosine residues that could be differentially sulfated, thereby generating up to eight ligands with distinct sulfation states (45). Importantly, the two receptor subtypes, apCCKR1 and apCCKR2, exhibit differential sensitivity to sulfation number (45), a pattern distinct from the currently characterized dual-sulfated systems such as those in *Magallana gigas* and *Ciona intestinalis* (40, 48). Specifically, apCCKR1 responds similar to monosulfated ligands ([sY8]-CCK1, [sY5]-CCK2) and disulfated ligands ([sY6,sY8]-CCK1, [sY2,sY5]-CCK2), whereas apCCKR2 favors the monosulfated forms. This divergence provides a tractable system for examining how related receptors decode both peptide C-terminal chemistry and sulfation state, and whether such recognition reflects ancient convergent principles, lineage-specific divergence, or both.

To address these questions mechanistically, however, a reliable molecular framework was required. Our initial modeling of apCCK system yielded conflicting binding poses, including both N-terminal-in and C-terminal-in orientations. Here, pose-specific mutagenesis was used to resolve this ambiguity to establish a common C-terminal-in binding mode for apCCK. Building on this experimentally validated framework, we identified a hierarchical mechanism for recognition of the aromatic C-terminus mediated by a three-residue motif, comprising a deeply conserved anchor residue, a CCK-biased interaction layer, and a flexible tuning site. We further uncovered a mechanism of sulfation-number-dependent subtype selectivity, in which the two apCCKRs respond differently to an additional sulfate through distinct electrostatic solutions. Specifically, an evolutionarily conserved charged core (arginine (R)-glutamate (E) pair) in the second extracellular loop (ECL2) supports sulfate recognition, while a lineage-restricted lysine (K) in transmembrane helix 7 (TM7) modulates subtype-specific responses. These findings enable us to propose an evolutionary comparative framework for how sulfation recognition has been conserved and remodeled across CCK receptors from representative protostome and deuterostome lineages.

Collectively, this study not only defines a binding mode in a single protostome system but also provides a molecular insight and a comparative framework for understanding how homologous CCK-family receptors preserve, remodel, or reassign recognition of conserved peptide chemical features. These findings highlight how conserved structural logic and adaptive diversification jointly shape ligand recognition, revealing both shared constraints and flexible solutions during the co-evolution of neuropeptides and their receptors.

## Results

### Mutagenesis-guided discrimination of docking poses reveals a C-terminal-in binding mode for *Aplysia* CCK peptides

Our prior work has established a complete CCK signaling system in *A. californica*, comprising two receptors, apCCKR1 and apCCKR2, together with multiple mature CCK peptides (45). To define the molecular basis of ligand recognition in this system, we first generated three-dimensional models of both receptors *via* the Swiss-Model and Robetta servers (Figs. S1 and S2), followed by structural comparison and validation with multiple quality indices (Fig. S3). We then selected the dual-sulfated [sY6,sY8]-CCK1 as a representative ligand for molecular docking.

Initial docking attempts using Discovery Studio, with the binding region defined by conserved G-protein coupled receptor (GPCR) active-site features, failed to yield a convincing binding solution. The resulting poses either failed to place the ligand within the receptor pocket or generated pocket-bound conformations that were exclusively N-terminal-in. Since this orientation is inconsistent with the C-terminally engaged binding mode reported in human CCK-CCKR complexes resolved by cryo-EM (41, 42, 43) (Fig. S4), we evaluated alternative docking approaches that may be more suitable. However, AutoDock (49) and diffdock (50) likewise failed to generate a plausible ligand-protein complex, suggesting that docking strategies commonly used for small molecules may be insufficient to reliably resolve the binding mode of the long [sY6,sY8]-CCK1 carrying unique sulfation modifications.

**Figure 1:**
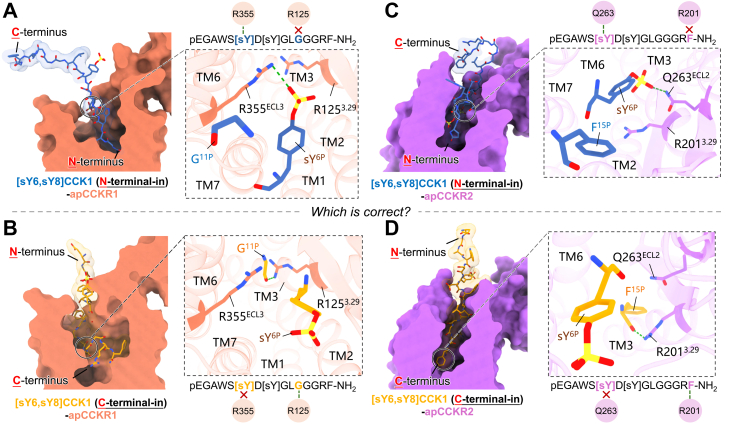
**Representative N-terminal-in and C-terminal-in docking models of [sY6,sY8]-CCK1 bound to apCCKR1 and apCCKR2, highlighting pose-specific residues and interactions.***A*, N-terminal-in model of [sY6,sY8]-CCK1 bound to apCCKR1, shown as a cutting-side view and an enlarged view of the circled region. The enlarged view shows the pose-specific residue R355^ECL3^ in the apCCKR1 N-terminal-in model and its hydrogen bond (*green dashed lines*) with the sulfate group of ligand sY^6^^P^. In the N-terminal-in model, [sY6,sY8]-CCK1 is shown as *blue* sticks and surface, and apCCKR1 is shown as *orange* surface, cartoon, and sticks. In all panels, nitrogen, oxygen, and sulfur atoms are *colored dark blue*, *red*, and *light yellow*, respectively. *B*, C-terminal-in model of [sY6,sY8]-CCK1 bound to apCCKR1, shown as a cutting-side view and an enlarged view of the circled region. The enlarged view shows the pose-specific residue R125^3.29^ in the apCCKR1 C-terminal-in model and its hydrogen bond with ligand G^11^^P^. R125^3.29^ also interacts with F^15^^P^, which is not shown in this panel; the details are presented in the next part. In the C-terminal-in model, [sY6,sY8]-CCK1 is shown as yellow sticks and surface. *C*, N-terminal-in model of [sY6,sY8]-CCK1 bound to apCCKR2, shown as a cutting-side view and an enlarged view of the circled region. The enlarged view shows the pose-specific residue Q263^ECL2^ in the apCCKR2 N-terminal-in model and its hydrogen bond with ligand sY^6^^P^. apCCKR2 is shown as *purple* surface, cartoon, and sticks. *D*, C-terminal-in model of [sY6,sY8]-CCK1 bound to apCCKR2, shown as a cutting-side view and an enlarged view of the circled region. The enlarged view shows the pose-specific residue R201^3.29^ in the apCCKR2 C-terminal-in model and its hydrogen bond with ligand F^15^^P^. These pose-specific residues and interactions provided testable criteria for discriminating between the two classes of competing models. In the superscripts of ligand residues shown in the panels, “P” denotes “peptide”. apCCKR, *Aplysia* cholecystokinin receptor.

We therefore employed HPEPDOCK, a platform optimized for peptide–protein docking (51). Notably, blind docking with HPEPDOCK yields two classes of pocket-bound models: ∼80% adopted an N-terminal-in pose, whereas the remaining 20% display a C-terminal-in configuration. Importantly, the N-terminal-in models could not be excluded *a priori*, because resolved structures from other GPCRs have shown that peptides do not invariably engage their receptors through C-terminal insertion (52, 53, 54) (see details in the Discussion section). Notably, the two classes of models shared many contacting receptor residues, even though the corresponding ligand-facing interactions could differ (Table S1). Therefore, the key to resolving such a model ambiguity was to focus on those sites that were unique to one pose and absent from the other. Thus, we compared different models and searched for pose-specific ligand-receptor interactions that are experimentally testable. Specifically, in apCCKR1, the N-terminal-in model predicted a specific hydrogen bond between the sulfate group of sY^6^ in [sY6,sY8]-CCK1 and receptor residue R355^ECL3^ (Fig. 1*A*), whereas this interaction was absent from the C-terminal-in model (Fig. 1*B*). By contrast, the C-terminal-in model also predicted a specific bonding between G^11^/F^15^ and R125^3.29^ (Fig. 1*B*), which was not observed in the N-terminal-in model (Fig. 1*A*). Similarly, in apCCKR2, the N-terminal-in model predicted a distinct sY^6^-Q263^ECL2^ interaction (Fig. 1*C*), whereas its counterpart predicted a distinct F^15^-R201^3.29^ interaction (Fig. 1*D*). These mutually exclusive contacts provided a direct experimental framework for model discrimination.

Throughout, we used the following notations to indicate residue locations in the receptor or ligand: superscripts on receptor residues denote either extracellular loop (ECL) assignment or Ballesteros-Weinstein numbering, in which the first number identifies the transmembrane helix and the second indicates the position relative to the conserved residue 50 (55). Peptide residues are indicated by sequence position, and sY denotes sulfated tyrosine.

Subsequently, we generated R355A and R125A mutants in apCCKR1 and Q263A and R201A mutants in apCCKR2, and examined their responses to [sY6,sY8]-CCK1 using the IP1 (inositol monophosphate) accumulation assay. To minimize the influence of potentially different endogenous G-protein coupling preferences, both wild-type and mutants were co-transfected with a promiscuous Gαq-family protein, which can couple a broad range of GPCRs to the inositol trisphosphate (IP3) signaling pathway and thereby provide a common functional readout using the IP1 accumulation assay (56).

A total of eight putative apCCKs have been identified, seven of which have been verified with mass spectrometry except for [sY2,sY5]-CCK2 (45). For functional assays, we focused on [sY8]-CCK1 and [sY6,sY8]-CCK1 as the representative mono- and disulfated ligands and included [sY5]-CCK2 due to its high potency at apCCKR2 (sub-100 nM EC_50_). However, all subsequent docking analyses were performed using [sY8]-CCK1 and [sY6,sY8]-CCK1 as a matched ligand pair.

**Figure 2:**
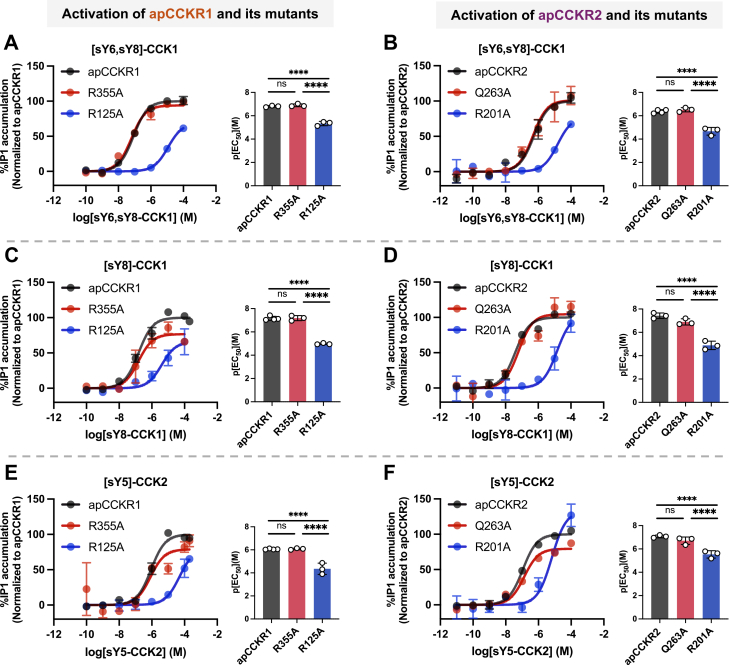
**Mutagenesis of pose-specific residues in the N-terminal-in and C-terminal-in models using [sY6,sY8]-CCK1 and two monosulfated apCCKs.***A, C, E*, representative dose-response curves showing the activation of apCCKR1 and its mutants (R355A and R125A) by [sY6,sY8]-CCK1 (*A*), [sY8]-CCK1 (*C*) and [sY5]-CCK2 (*E*). All curves were normalized to 100% of the maximal response induced by the corresponding peptide in wild-type apCCKR1. The bar graphs summarize the pEC_50_ values for [sY6,sY8]-CCK1 (*A*), [sY8]-CCK1 (*C*) and [sY5]-CCK2 (*E*) at apCCKR1 and its two mutants. For *panel* (*A*), n ≥ 3. One-way ANOVA, F (2, 7) = 181.5, *p* < 0.0001. For *panel* (*C*), n ≥ 3. One-way ANOVA, F (2, 6) = 130.4, *p* < 0.0001. For *panel* (*E*), n ≥ 3. One-way ANOVA, *F* (2, 7) = 42.93, *p* = 0.0001. Tukey *post hoc* test: n.s., not significant; ∗∗∗∗*p* < 0.0001. Error bar: SD. *B, D, F,* representative dose-response curves showing the activation of apCCKR2 and its mutants (Q263A and R201A) by [sY6,sY8]-CCK1 (*B*), [sY8]-CCK1 (*D*) and [sY5]-CCK2 (*F*). All curves were normalized to 100% of the maximal response induced by the corresponding peptide in wild-type apCCKR2. The bar graphs summarize the pEC_50_ values for [sY6,sY8]-CCK1 (*B*), [sY8]-CCK1 (*D*) and [sY5]-CCK2 (*F*) at apCCKR2 and its two mutants. For *panel* (*B*), n ≥ 3. One-way ANOVA, F (2, 7) = 64.75, *p* < 0.0001. For panel (*D*), n = 3. One-way ANOVA, F (2, 6) = 62.11, *p* < 0.0001. For panel (*F*), n = 3. One-way ANOVA, F (2, 6) = 27.43, *p* = 0.001. Tukey *post hoc* test: n.s., not significant; ∗∗∗∗*p* < 0.0001. Error bar: SD. Across all mutagenesis, only mutations of the C-terminal-in pose-specific residues significantly altered ligand potency. p[EC_50_], -log_10_(EC_50_).

For [sY6,sY8]-CCK1, in apCCKR1, R355A did not affect receptor activation, whereas R125A markedly reduced ligand potency (Fig. 2*A* and Table S2). Likewise, in apCCKR2, Q263A produced little detectable effect, whereas R201A significantly impaired receptor activation (Fig. 2*B* and Table S2). Both [sY8]-CCK1 and [sY5]-CCK2 recapitulated the mutational sensitivity profile observed for [sY6,sY8]-CCK1 (Fig. 2, *C*–*F* and Table S2). Cell surface expression analyses of FLAG-tagged receptor mutants showed levels comparable to wild-type receptors (Table S2), indicating that the observed changes in EC_50_ were unlikely to result from receptor expression or trafficking.

Collectively, in both receptors, the residues functionally required for apCCKs recognition were those uniquely predicted by the C-terminal-in model, whereas those selected from the N-terminal-in model were dispensable, supporting a common C-terminally engaged binding mode for the apCCK peptides.

### Recognition of amidated aromatic C-terminus (RFamide/DFamide) reveals conserved and divergent decoding in CCK receptors

Having established a reliable C-terminal-in binding model for the apCCK signaling system, we next turned to the broader question: how conserved peptide chemical features are recognized by receptors across evolution, and to what extent such recognition is preserved or diversified.

**Figure 3:**
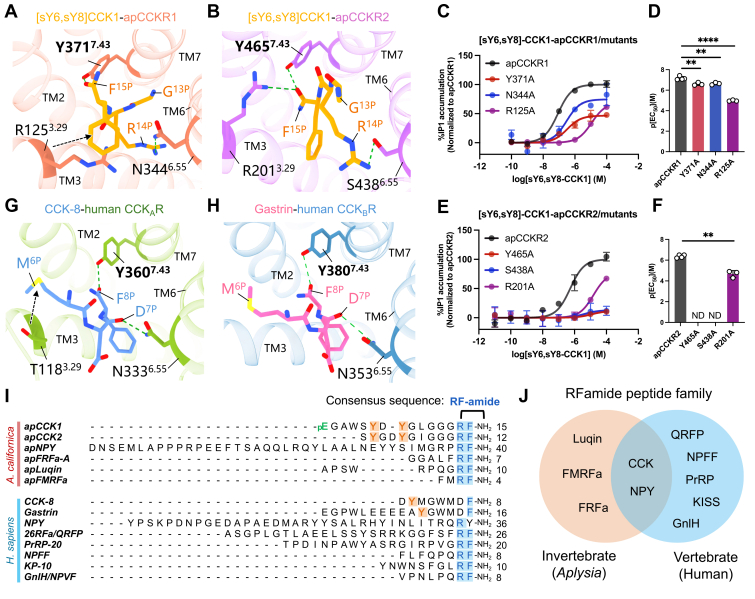
**Conserved and divergent receptor-side decoding of the C-terminal RF/DFamide motif in *Aplysia* and human CCK receptors.***A*, representative C-terminal-in model of [sY6,sY8]-CCK1 bound to apCCKR1. Only the peptide residues G^13^-R^14^-F^15^ are shown as yellow sticks, whereas the receptor is shown as *orange* cartoon and sticks. *B*, representative C-terminal-in model of [sY6,sY8]-CCK1 bound to apCCKR2. Receptor is shown as *purple* cartoon and sticks. In panels (*A-B*, *G-H*), nitrogen, oxygen, and sulfur atoms are colored *dark blue*, *red*, and *light yellow*, respectively. Hydrogen bonds and hydrophobic interactions are indicated by *greendashed lines*. *C*, representative dose-response curves showing activation of apCCKR1 and its mutants (Y371A, N344A and R125A) by [sY6,sY8]-CCK1. All curves were normalized to 100% of the maximal response induced by [sY6,sY8]-CCK1 in wild-type apCCKR1. *D*, Bar graphs summarizing the pEC_50_ values for [sY6,sY8]-CCK1 at wild-type apCCKR1 and the three mutants. n ≥ 3. One-way ANOVA, F (3, 9) = 155.0, *p* < 0.0001. *E*, representative dose-response curves showing activation of apCCKR2 and its mutants (Y465A, S438A and R201A) by [sY6,sY8]-CCK1. All curves were normalized to 100% of the maximal response induced by [sY6,sY8]-CCK1 in wild-type apCCKR2. *F*, Bar graphs summarizing the pEC_50_ values for [sY6,sY8]-CCK1 at wild-type apCCKR2 and the three mutants. n ≥ 3. ND, not detected. Paired two-tailed *t* test: ∗∗*p* < 0.01; ∗∗∗∗*p* < 0.0001. Error bars indicate SD. The activation curves and data of the R125A and R201A mutants have also been presented in Figure 2. *G*, structural model of the CCK-8-human CCK_A_R complex (PDB: 7EZH) (41). Only the peptide residues M^6^-D^7^-F^8^ are shown as *blue* sticks, whereas the receptor is shown as *green* cartoon and sticks. *H*, Structural model of the gastrin-human CCK_B_R complex (PDB: 7F8V) (42). Only the peptide residues M^6^-D^7^-F^8^ are shown as *pink sticks*, whereas the receptor is shown as *blue* cartoon and sticks. S^3.29^ does not contact CCK_B_R and is therefore not shown in this panel. Panels (*A*-*B*) and (*G*-*H*) display views of the deep regions within the binding pockets. *I*, sequence alignment of representative RFamide peptides from invertebrates (using *Aplysia* as a representative) and vertebrates. The N-terminal E of apCCK1, which can undergo pyroglutamylation, is highlighted in bold *green*. Sulfated tyrosines in apCCKs and in human CCK-8/gastrin are highlighted in *yellow*. Conserved C-terminal RFamide residues are highlighted in *blue*. In vertebrates, NPY terminates in RYamide, whereas CCK-8/gastrin terminates in DFamide. *J*, Venn diagram showing the distribution of RFamide peptide families in invertebrates and vertebrates. CCK and NPY are shared between the two groups. NPY, neuropeptide Y; 26RFa/QRFP, pyroglutamylated RFamide peptide-26; PrRP-20, prolactin-releasing peptide-20; NPFF, neuropeptide FF; KP-10, kisspeptin-10; GnIH, gonadotropin-inhibitory hormone; NPVF, neuropeptide VF.

In the *Aplysia* system, apCCK peptides contain a C-terminal RFamide motif. Using [sY6,sY8]-CCK1 as the representative ligand, we first asked which receptor-side residues support recognition of this region. In apCCKR1, docking predicted that Y371^7.43^ formed a hydrogen bond with peptide F^15^, N344^6.55^ interacted with R^14^, and R125^3.29^ contacted F^15^ through hydrophobic interaction (Fig. 3*A*). In apCCKR2, the basic framework involving positions 7.43 and 6.55 was likewise retained, with Y465^7.43^ engaging F^15^ and S438^6.55^ contacting F^15^. However, in this receptor, R201^3.29^ was predicted to form a hydrogen bond with F^15^ rather than a hydrophobic contact (Fig. 3*B*).

Functional validation by mutagenesis further supported these predictions. In apCCKR1, Y371A, N344A and R125A significantly reduced the potency of [sY6,sY8]-CCK1 (Fig. 3, *C* and *D* and Table S2). In apCCKR2, R201A also produced significant effects, while Y465A and S438A abolished detectable ligand-induced IP1 accumulation across the tested concentration range, indicating that these two orthosteric residues are essential for productive ligand-induced receptor activation. Although the absence of activity in the two mutant receptors is most consistent with impaired ligand binding, contributions from altered G protein coupling cannot be excluded (Fig. 3, *E* and *F* and Table S2). Notably, R^3.29^ (R125 in apCCKR1, R201 in apCCKR2) has already emerged as a key determinant during docking-pose discrimination (Fig. 2). Consistent results were obtained with [sY8]-CCK1 and [sY5]-CCK2, both of which exhibited similar sensitivity to the same mutations (Fig. S5 and Table S2). These data showed that positions 7.43, 6.55, and 3.29 collectively contribute to receptor-side recognition of the C-terminal RFamide in *Aplysia* CCK receptors, and that the two subtypes appeared to use a shared topological framework but differ in the local chemistry of C-terminal recognition.

We then compared this *Aplysia*-derived recognition pattern with high-resolution cryo-EM structures of human CCK receptors, whose cognate CCK/gastrin peptides terminate in a DFamide motif. In the CCK-8-CCK_A_R complex (PDB: 7EZH) (41), Y360^7.43^ firmly anchors the peptide F^8^, N333^6.55^ interacts with D^7^, and T118^3.29^ tends to participate in a hydrophobic force with the M^6^ immediately next to the DFamide motif (Fig. 3*G*). This pattern resembles apCCKR1 in that all three positions contribute to the local recognition network. By contrast, in the gastrin-CCK_B_R complex (PDB: 7F8V) (42), the interactions mediated by Y380^7.43^ and N^6.55^ are retained, whereas position 3.29 is occupied by serine (S) and does not contribute detectably to ligand recognition (Fig. 3*H*). Thus, the human structures support the same overall hierarchy inferred from the *Aplysia* system despite some divergence.

Given that CCK-family peptides terminate in RFamide in protostomes but DFamide in most deuterostomes, we further asked whether the receptor-side recognition motif identified above could extend beyond the CCK signaling system to the broader RFamide-family context (32, 36) (Fig. 3, *I* and *J*, the representative CCK and RF amide peptides in *Aplysia* and human are shown in Fig. 3*I*). Analysis of representative human RFamide receptors, such as QRFPR, NPFFR and KISSR, revealed that residue 7.43 consistently engaged the ligand C-terminal F (44, 46). In cryo-EM ligand-receptor complexes for human NPY receptors, where the peptide C termini have diverged to RYamide (Fig. 3*I*), the Y likewise interacted with the conserved 7.43 through hydrophobic interaction (17). Then, we performed BioEdit comparisons of all predicted *Aplysia* RFamide receptors (Figs. S6, S7 and Table S3) and a preliminary docking of the C-terminal interactions of all systems by known ligands and these receptors (57, 58, 59, 60, 61) (Fig. S8). Strikingly, the same general pattern was observed: residue 7.43 engaged the ligand F across multiple *Aplysia* RFamide systems. Although the precise residue identity of 7.43 (mostly hydrophobic property) or its contact chemistry (Y-hydrogen bond, M, F-hydrophobic interaction) varies among receptors, they all contact F in the ligand and are therefore strongly conserved. By contrast, other than CCK receptors in *Aplysia* and humans, no clear functional role was evident for positions 6.55 and 3.29 across the other RFamide families examined here, suggesting that these two sites may be less broadly conserved and more family- or system-specific than 7.43.

### Sulfation-sensitive interaction underlies subtype-selective activation of *Aplysia* CCK receptors

In addition to the conserved C-terminal motif, CCK-family peptides contain a second key PTM feature, tyrosine sulfation, which is structurally and evolutionarily more complicated. The above clear definition of RFamide recognition in the C-terminal-in models provided a framework for addressing the next question. Our previous study has demonstrated that tyrosine sulfation is essential for receptor activation and physiological actions of *Aplysia* CCK peptides, and the two receptor subtypes respond differently to CCKs carrying different numbers of sulfate groups (45). Specifically, in apCCKR1, monosulfated peptides including [sY8]-CCK1 and [sY5]-CCK2 displayed potencies comparable to those of their dual-sulfated counterparts, [sY6,sY8]-CCK1 and [sY2,sY5]-CCK2. In contrast, in apCCKR2, monosulfated CCKs were consistently more potent than the corresponding dual-sulfated ligands. However, the molecular basis for this receptor subtype selectivity (45) is unknown.

**Figure 4:**
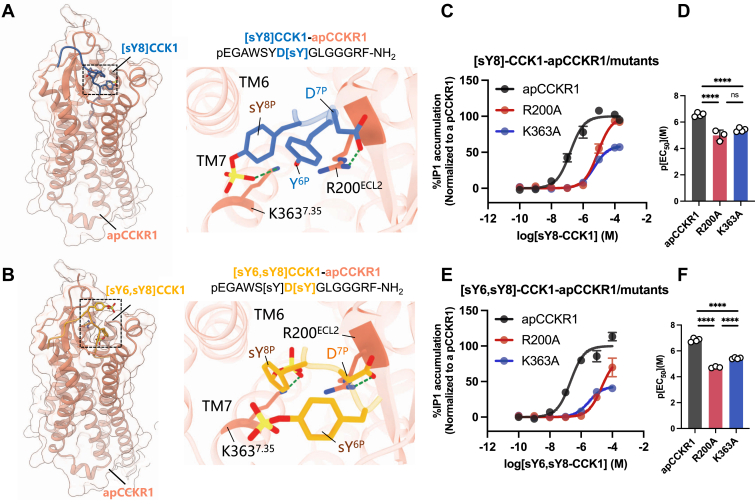
**Molecular basis for similar activation of apCCKR1 by mono- and di-sulfated apCCK1 peptides.***A*, overall and zoomed-in views of the C-terminal-in docking model of [sY8]-CCK1 bound to apCCKR1. The *right panel* shows an enlarged view of the boxed region in the overall receptor structure. Only the Y^6^-D^7^-sY^8^ region of the peptide is shown as *blue* sticks, whereas key receptor residues are shown as *orange* cartoon and sticks. *B*, overall and zoomed-in views of the C-terminal-in docking model of [sY6,sY8]-CCK1 bound to apCCKR1. Only the sY^6^-D^7^-sY^8^ region of the peptide is shown as *yellow* sticks. Nitrogen, oxygen, and sulfur atoms are colored *dark blue*, *red*, and *light yellow*, respectively. Salt bridges are indicated by *greendashed lines*. *C*, representative dose-response curves showing the activation of apCCKR1 and its mutants (R200A and K363A) by [sY8]-CCK1. All curves were normalized to 100% of the maximal response induced by [sY8]-CCK1 in wild-type apCCKR1. *D*, Bar graphs summarizing the pEC_50_ values for [sY8]-CCK1 at apCCKR1 and its two mutants. n ≥ 3. One-way ANOVA, F (2, 8) = 41.80, *p* < 0.0001. *E*, representative dose-response curves showing the activation of apCCKR1 and its mutants (R200A and K363A) by [sY6,sY8]-CCK1. All curves were normalized to 100% of the maximal response induced by [sY6,sY8]-CCK1 in wild-type apCCKR1. *F*, Bar graphs summarizing the pEC_50_ values for [sY6,sY8]-CCK1 at apCCKR1 and its two mutants. n ≥ 3. One-way ANOVA, F (2, 8) = 417.5, *p* < 0.0001. Tukey *post hoc* test: n.s., not significant; ∗∗∗∗*p* < 0.0001. Error bar: SD.

To elucidate the structural basis of this divergence, we compared docking models of [sY8]-CCK1 and [sY6,sY8]-CCK1 in both receptors. Across all modeled configurations, residue Y^6^/sY^6^ did not participate directly in receptor-side recognition, whereas Y^8^/sY^8^ consistently emerged as the principal sulfate-bearing determinant, which aligns with the activation pattern established previously (45). In apCCKR1, D^7^ of [sY8]-CCK1 formed a salt bridge with receptor residue R200^ECL2^, and the sulfate group of sY^8^ salt-bridged with K363^7.35^ (Fig. 4*A*). Notably, the dual-sulfated [sY6,sY8]-CCK1 adopted a nearly identical interaction pattern, indicating that addition of the second sulfate does not substantially alter the mode of receptor engagement in apCCKR1 (Fig. 4*B*). Consistent with this model, mutagenesis and functional assays showed that both R200A and K363A significantly reduced ligand potency for both mono- and dual-sulfated ligands (Fig. 4, *C*–*F* and Table S2).

**Figure 5:**
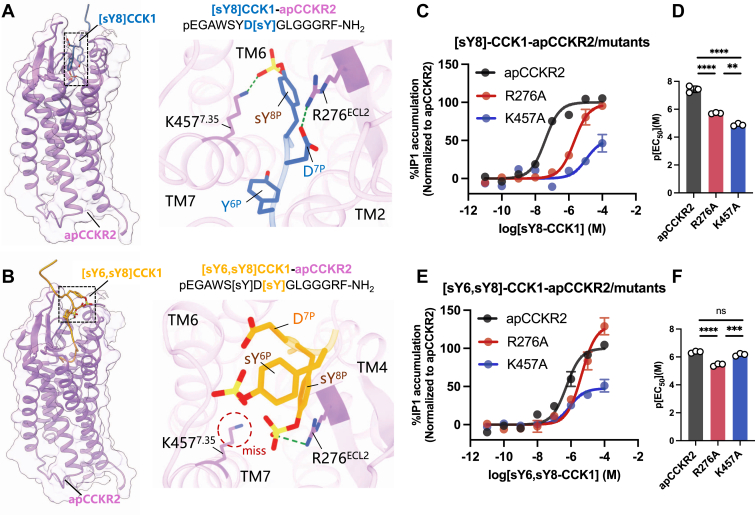
**Molecular basis for the higher potency of monosulfated *versus* disulfated apCCK1 peptides at apCCKR2.***A*, overall and zoomed-in views of the C-terminal-in docking model of [sY8]-CCK1 bound to apCCKR2. The *right panel* shows an enlarged view of the boxed region in the overall receptor structure. Only the Y^6^-D^7^-sY^8^ region of the peptide is shown as *blue* sticks, whereas key receptor residues are shown as *purple* cartoon and sticks. *B*, overall and zoomed-in views of the C-terminal-in docking model of [sY6,sY8]-CCK1 bound to apCCKR2. Only the sY^6^-D^7^-sY^8^ region of the peptide is shown as *yellow* sticks. Nitrogen, oxygen, and sulfur atoms are colored *dark blue*, *red*, and *light yellow*, respectively. Salt bridges are indicated by *greendashed lines*. *C*, Representative dose-response curves showing the activation of apCCKR2 and its mutants (R276A and K457A) by [sY8]-CCK1. All curves were normalized to 100% of the maximal response induced by [sY8]-CCK1 in wild-type apCCKR2. *D*, bar graphs summarizing the pEC_50_ values for [sY8]-CCK1 at apCCKR2 and its two mutants. n = 3. One-way ANOVA, F (2, 6) = 227.0, *p* < 0.0001. *E*, representative dose-response curves showing the activation of apCCKR2 and its mutants (R276A and K457A) by [sY6,sY8]-CCK1. All curves were normalized to 100% of the maximal response induced by [sY6,sY8]-CCK1 in wild-type apCCKR2. *F*, Bar graphs summarizing the pEC_50_ values for [sY6,sY8]-CCK1 at apCCKR2 and its two mutants. n = 3. One-way ANOVA, F (2, 6) = 86.27, *p* < 0.0001. Tukey *post hoc* test: n.s., not significant; ∗∗*p* < 0.01; ∗∗∗*p* < 0.001; ∗∗∗∗*p* < 0.0001; Error bar: SD.

In apCCKR2, D^7^ of [sY8]-CCK1 interacted with R276^ECL2^, while the sulfate sY^8^ engaged K457^7.35^ (Fig. 5*A*). In the [sY6,sY8]-CCK1 model, however, this arrangement was reconfigured: the interaction involving R276^ECL2^ was altered to contact the sulfate sY^8^, and K457^7.35^ no longer participated in ligand binding (Fig. 5*B*). Thus, unlike apCCKR1, apCCKR2 did not passively accommodate the second sulfate; instead, the additional modification disrupted the electrostatic network favored by the monosulfated ligand. Functional analyses supported this model. Mutation of K457^7.35^ significantly impaired receptor activation by [sY8]-CCK1 (Fig. 5, *C* and *D* and Table S2), but had little effect on [sY6,sY8]-CCK1 (Fig. 5, *E* and *F* and Table S2), whereas mutation of R276^ECL2^ remarkably affected receptor activation in both cases (Fig. 5, *C*–*F* and Table S2).

**Figure 6:**
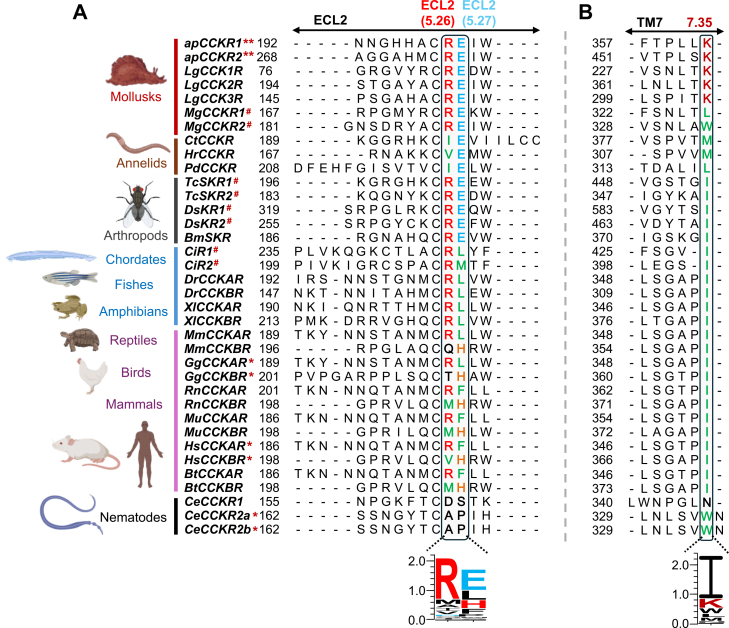
**Sequence alignment reveals topologically equivalent residues underlying subtype selectivity in apCCKRs and their comparative patterns across lineages.***A*, conservation and divergence of two key residues in the ECL2/TM5-adjacent region of apCCK receptors from different species. For consistency across receptors, these two positions were designated ECL2 (5.26) and ECL2 (5.27) according to the Ballesteros-Weinstein numbering system (55) instead of ECL2 as designated in the Results. At position ECL2 (5.26), R is highlighted in *red* bold; at position ECL (5.27), E is highlighted in *blue* bold and H in *orange* bold. Other hydrophobic residues are shown in *green* bold. The ECL2 (5.26)-ECL2 (5.27) pattern of nematode CCKR-like sequences does not follow the lineage-dependent trend observed in other groups and is therefore shown without additional color emphasis. Note the little conservation with ECL2 (5.26)-ECL2 (5.27) in *C. elegans* receptors, likely due to the absence of tyrosine in their CCK ligands. *B*, lineage-dependent variation at residue 7.35 in the TM7 region of apCCK receptors from different species. K is highlighted in *dark red bold*, whereas other residues with hydrophobicity are shown in *green bold*. The consensus shown below summarizes the residue frequency at the corresponding positions. ∗∗ indicates CCKR subtype-selective activation by sulfation number, in which the two receptor subtypes respond differently to mono-*versus* disulfated ligands. This pattern has been found only in *Aplysia* and may potentially occur in *Lottia*; ∗ indicates CCKR subtype-selective decoding of sulfation state, in which one subtype shows a stronger preference for sulfated CCK whereas the other discriminates weakly. This pattern has been demonstrated only in post-reptiles; # indicates no evident receptor-subtype divergence in sulfation recognition, that is, both receptor subtypes show stronger activation by sulfated ligands. This pattern has been demonstrated only in sampled non-amniote lineages. ap, *Aplysia californica*; Lg, *Lottia gigantea*; Mg, *Magallana gigas*; CtCCKR, *Capitella teleta*; Hr, *Helobdella robusta*; Pd, *Platynereis dumerilii*; Tc, *Tribolium castaneum*; Ds, *Drosophila melanogaster*; Bm, *Bombyx mori*. Ci, *Ciona intestinalis*; Dr, *Danio rerio*; Xl, *Xenopus laevis*; Mm, *Mauremys mutica*; Gg, *Gallus gallus*; Rn, *Rattus norvegicus*; Mu, *Mus musculus*; Hs, *Homo sapiens*; Bt, *Bos taurus*; Ce, *Caenorhabditis elegans*; ECL, Extracellular loop; TM, transmembrane domain.

Interestingly, the residues mediating these interactions occupy topologically equivalent positions in the two receptor subtypes. In apCCKR1 and apCCKR2, R200/R276^ECL2^ and K363/K457^7.35^ align structurally (Figs. 6 and S9) and perform analogous electrostatic roles. This raised a broader question: whether these sites are evolutionarily conserved across the CCK receptor family, or do they represent lineage- or subtype-specific solutions that emerged during receptor diversification?

To address this, we examined a broad set of identified and predicted CCK receptor sequences across diverse species and compared the regions corresponding to the residues implicated above (Complete sequence alignment is shown in Fig. S9, and the sequence sources are listed in Table S4). Our analysis was initially centered on R^ECL2^. Sequence alignment showed that R^ECL2^ and its adjacent E^ECL2^ displayed clear lineage-dependent patterns. For clarity and for more consistent comparison across receptors from different species, we therefore mapped these two neighboring residues onto Ballesteros-Weinstein numbering (55) and here operationally designated them as R^ECL2 (5.26)^-E^ECL2 (5.27)^ to distinguish the two residues (Fig. 6*A*). In invertebrate CCK receptors, especially in mollusks and arthropods, these two residues were strongly conserved (Fig. 6*A*). In annelids, the basic R^ECL2 (5.26)^ was replaced by hydrophobic residues such as I/V, whereas the adjacent E^ECL2 (5.27)^ was conserved (Fig. 6*A*). In vertebrates, further diversification was observed: chordates, fish, and amphibians generally retained R^ECL2 (5.26)^, whereas hydrophobic M/L replaced E^5.27^ (Fig. 6*A*). Most intriguingly, from reptiles and birds onward, these two residues diverge at the receptor subtype level. In CCK_A_R-like receptors, R^ECL2 (5.26)^ was conserved, whereas position ECL2 (5.27) was occupied by hydrophobic residues like L/F. In CCK_B_R-like receptors, position ECL2 (5.26) diverged to Q/T/M/V, accompanied by a fixed H at ECL2 (5.27). This subtype-specific combination was then maintained throughout mammals, including human CCKRs. By contrast, nematode CCKR-like sequences did not exhibit any recognizable similarity at these two positions (Fig. 6*A*), a divergence that may be related to the loss of tyrosine in their CCK-like ligands (62).

In contrast to the broad but patterned variation of R^ECL2 (5.26)^-E^ECL2 (5.27)^, the residue corresponding to K^7.35^ shows greater lineage-dependent variation (Fig. 6*B*). Among the receptors examined, the lysine was detected only in two mollusks, *that is, Aplysia* and L. *gigantea* with three predicted CCK receptors. In all other species analyzed, the equivalent site was occupied by hydrophobic residues with markedly different physicochemical properties, including L, W, M or F. Notably, in the molluscan *M. gigas* (previously *C. gigas*) receptors, this positively charged K is absent (Fig. 6*B*) even though one of its CCK ligands does have two tyrosines (40).

**Figure 7:**
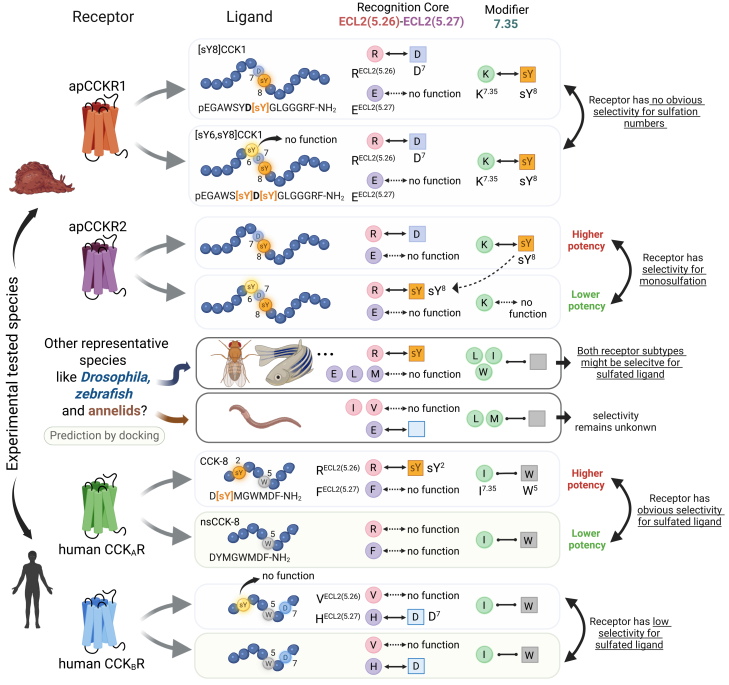
**Comparative models of conserved and lineage-specific sulfation recognition mechanisms across *Aplysia* and human CCK receptors with inferred patterns in other lineages.** Based on the analysis in Figure 6, an R^ECL2 (5.26)^-E^ECL2 (5.27)^-recognition core together with a K^7.35^ modifier is proposed to jointly determine sulfation recognition and receptor subtype selectivity. In apCCKR1, both monosulfated and disulfated ligands adopt a similar overall recognition mode: R^ECL2 (5.26)^ interacts with D^7^ in the peptide, K^7.35^ engages the sulfate group of sY^8^, and E^ECL2 (5.27)^ does not contribute detectably. Accordingly, apCCKR1 shows little selectivity for sulfation number. In apCCKR2, the monosulfated ligand follows a similar pattern as apCCKR1, but the introduction of a second sulfation does not generate an additional direct interaction by sY^6^ itself; instead, it induces a conformational reconfiguration in which sY^8^ shifts from K^7.35^ to R^ECL2 (5.26)^, leading to subtype-specific activation differences. In human CCK_A_R, sulfation is primarily recognized by R^ECL2 (5.26)^, whereas 7.35 is occupied by a hydrophobic I and interacts with W^5^. Residue E^ECL2 (5.27)^, despite changing to F, remains functionally silent. Although the detailed binding mode of nonsulfated CCK has not been structurally resolved, the absence of sulfation is expected to disrupt the R^ECL2 (5.26)^-mediated interaction, consistent with the well-known sulfation dependence of CCK_A_R activation. In human CCK_B_R, residues ECL2 (5.26) and ECL2(5.27) are replaced by V and H, respectively, and sulfate recognition is diminished; instead, H^ECL2 (5.27)^ contributes to recognition of the D^7^, while I^7.35^ maintains hydrophobic interactions. Accordingly, CCK_B_R shows relatively weak selectivity for sulfation. For other representative extant lineages lacking direct experimental characterization, the model predicts distinct receptor-side recognition patterns. In *M. gigas*, *Drosophila*, zebrafish, and *C. intestinalis*, both receptor subtypes are proposed to retain R-mediated sulfate recognition at 5.26 (and indeed, we also demonstrated this in our receptor docking for *Drosophila*, as shown in Fig. S11), while L/I/W^7.35^ contributes hydrophobic support, suggesting limited subtype divergence in sulfation decoding. In annelids, substitution of R^ECL2 (5.26)^ by I/V is predicted to abolish direct sulfate recognition (see Fig. S12 for *Capitella* and *Helobdella* docking), whereas E^ECL2 (5.27)^ may assume alternative roles in ligand interaction; residues at L/M^7.35^ are predicted to maintain hydrophobic support. In this schematic, solid double-headed arrows indicate salt bridges, dashed arrows indicate the absence of detectable interaction, and paired filled circles indicate hydrophobic interactions. Ligand residues are indicated as rectangles with different filled colors; receptor residues as circles with different filled colors. Circles with the letter “sY” in the ligands indicate sulfated tyrosine, with functionally engaged sulfates shown in *orange* and non-interacting sulfates in *light yellow*.

Together, these results indicated that apCCKR1 preserves a stable interaction network across mono- and di-sulfated ligands, whereas apCCKR2 undergoes a sulfation-dependent reconfiguration of ligand-receptor contacts. More broadly, the residues underlying this subtype selectivity in *Aplysia* are not universally conserved across the CCK receptor family, suggesting that distinct receptor-side solutions have emerged across different lineages for recognizing peptide sulfation (Figs. 6 and 7) (see details in the Discussion section).

## Discussion

Leveraging the tractable *Aplysia* CCK system, this study integrated *in silico* docking, pose-specific mutagenesis, and structure-function analysis to address an ambiguity in neuropeptide-GPCR modeling. More broadly, our findings reveal both convergence and divergence in how homologous receptors recognize conserved peptide PTMs during ligand-receptor co-evolution.

### Pose-specific mutagenesis resolves docking ambiguity in neuropeptide-GPCR modeling

Our initial docking attempts yielded inconsistent and often implausible binding conformations, largely because we repeatedly applied a single approach while varying only the parameters. More plausible models only emerged after systematically expanding the search to include multiple docking methods. In our view, this reflects a general feature of neuropeptide-GPCR modeling: ligand-specific features, including peptide length, conformational flexibility, and complex PTMs, can strongly influence docking performance and bias the resulting conformational space (9, 11, 12). Consequently, no single computational framework is universally optimal, and different neuropeptide systems may require distinct modeling strategies (30). For example, whereas AutoDock proved informative in our previous study about *Aplysia* leucokinin signaling (31), it was inadequate for the present CCK system. Likewise, HPEPDOCK or AlphaFold-based approaches, despite their strengths, do not consistently yield reliable solutions across all neuropeptide-GPCR pairs. Together, these observations argue for a multi-method strategy as a practical standard when initial docking is inconclusive, which may not be the most suitable one.

A major and often underappreciated challenge in current GPCR docking lies in the correct ligand orientation or pose. Competing models, including N-terminal-in, C-terminal-in, or alternative noncanonical geometries, are not uncommon (63). Importantly, these poses should not automatically be taken as evidence of modeling failure. Recent studies have shown that ligands can engage GPCRs through markedly different modes. In the ghrelin-GHSR cryo-EM complex, the ligand indeed extends into the receptor pocket through its N-terminal region, and the study explicitly noted that this mode was totally inconsistent with its prior expectations (53). In galanin receptors, the peptide lies largely across the extracellular vestibule rather than adopting a simple N-terminal-in or C-terminal-in configuration (54). More broadly, class B GPCRs recognize their ligands through still more complex binding schemes (52).

Equally important, even when alanine-scanning mutagenesis confirms the functional importance of residues predicted from docking, this does not necessarily validate the overall pose. Competing models can also share partially overlapping contact residues, yet these receptor residues might interact with different residues in the ligand (Table S1). In such cases, limited mutagenesis may identify functionally important sites without truly discriminating among alternative geometries. This limitation highlights that it is critical to find the correct poses using the residues that are different.

In the *Aplysia* CCK system, we addressed this issue by clearly comparing candidate poses and selecting pose-specific residues, that is, residues predicted only in the N-terminal-in model or only in the C-terminal-in model (Fig. 1). Mutation of such residues provides substantially greater discriminatory power, as it allows experimental rejection of an entire class of models rather than merely failing to support a preferred one (Fig. 2 and Table S2). Using this framework, we were able to rule out the predominant N-terminal-in solutions and support a C-terminal-in binding mode for *Aplysia CCK* peptides without requiring exhaustive mutational screening. We therefore suggest that this pose-specific, experimentally guided strategy may provide a potentially generalizable approach for improving neuropeptide-GPCR model selection when high-resolution structural evidence is not yet available.

### From CCK to RFamide: a conserved anchoring and progressive divergence in ligand C-terminal recognition

RFamide-type peptides were first recognized in protostomes and comprise multiple neuropeptide families (64, 65, 66), including luqin (59), FMRFa (57), FRFa (60), NPF/NPY (58, 67), and sulfakinins/CCK (34, 45, 68, 69, 70). These peptides are also widespread in deuterostomes, where related families include QRFP (35), NPFF (71), PrRP (39), KISS (kisspeptin) (39), and GnIH/RFRP (39). While protostome NPF/NPY and CCK peptides retain canonical RFamide-type C termini, their vertebrate counterparts have undergone modifications, with NPY-family peptides terminating in RYamide (38) and CCK/gastrin peptides terminating in DFamide (33). Despite these variations, our comparative analyses treat them as RFamide-related systems that preserve a shared problem of receptor-side recognition of amidated aromatic C termini (Fig. 3, *I* and *J*). Interestingly, our data indicated that receptor-side decoding of this amidated aromatic C-terminus was not uniformly conserved, but organized hierarchically, with deeply conserved anchoring residues (7.43) layered with progressively more flexible, lineage and subtype-specific components (6.55 and 3.29).

At the core of this hierarchy, residue 7.43 (Y) emerges as a highly conserved anchoring site. In the CCK system, this position stabilizes the aromatic F at the peptide C-terminus and exhibits remarkable conservation across evolutionary distance. Notably, in both *Aplysia* and human CCK receptors, the residue type, interaction partner, and even interaction roles are all essentially unchanged (Fig. 3, *A*, *B*, *G* and *H*). This conservation is consistent with recent cryo-EM structures of human RFamide receptor (17, 44, 46) and with our modeled *Aplysia* RFamide complexes (Figs. 3, *A*, *B*, *G*, *H* and S8). Even in more divergent RFamide receptors, this position remains predominantly hydrophobic, supporting a conserved role in anchoring the peptide C-terminal aromatic side chain.

By comparison, residue 6.55 (mostly N) represents an intermediate layer of conservation. While its interaction logic is broadly stable in the CCK system, its chemical conservation is clearly weaker than at 7.43. Concerning conservation, this site in the human CCK receptors interacts with D at the peptide C-terminus, whereas in apCCKRs it retains the same interaction even though the corresponding peptide residue is R, a side chain with opposite charge properties (Fig. 3, *A*, *B*, *G* and *H*). With regard to divergence, this 6.55 residue is replaced by S (also contacting R) in apCCKR2 (Fig. 3*D*), and no consistent functional role for this position is evident from broader RFamide receptor comparisons (44) (Figs. S6 and S7). These observations suggest that 6.55 constitutes a CCK-specific layer of binding, which is more family-specific than universally RFamide-defining.

Differently, residue 3.29 is an evolutionarily plastic component of the recognition network. Across the four CCK receptors analyzed here, its residue identity, interaction partners and interaction modes vary substantially (Fig. 3, *A*, *B*, *G* and *H*), and no comparable role is observed across the broader RFamide-family survey (44) (Figs. S6 and S7). This variability indicates that 3.29 is not part of the conserved recognition core itself but instead serves as a peripheral tuning site that allows different receptor families and subtypes to implement the same peptide-recognition problem in distinct structural ways. Once the peptide C-terminus has been anchored by the conserved elements such as 7.43, positions like 3.29 can diversify to modulate subtype preference, stabilize local networks, or influence signaling efficiency in lineage-specific ways. Together, the three sites suggest a layered model for receptor-side decoding of peptide C-terminus features, with 7.43 as the anchor, 6.55 as a more family-biased selector, and 3.29 as a flexible evolutionary tuner.

Importantly, our findings also highlight limitations in both structural and computational approaches for identifying functionally critical residues. Specifically, in human CCK_B_R, examination of the available structures (41, 42, 43) showed that the CCK-8-CCK_B_R complex did not clearly resolve the critical Y353^7.43^ (43). For this reason, we used gastrin as the principal reference ligand for CCK_B_R-side comparison (42). It suggests that the CCK-8-CCK_B_R study might have missed this interaction; thus, even closely related complexes may differ in how clearly key interactions are resolved (15, 16). More broadly, previous docking studies without mutagenesis validation have occasionally implicated positions 7.43, 6.55, and 3.29 in *Drosophila* (72, 73), but no other RFamide or CCK signaling system, including *Tribolium castaneum* (74), has yet systematically resolved the coordinated roles of these three positions with comparable experimental support.

Our results underscore that both experimentally determined structures and computational models may overlook evolutionarily conserved functional features. In this study, the three-position motif was first functionally resolved in the *Aplysia* CCK system, and subsequent comparison with human CCKR structures revealed that the same topological layer is also embedded in mammalian CCK/gastrin recognition. Individual residues at these positions could be observed in human cryo-EM structures, fully in the CCK-8-CCK_A_R complex (41, 43) (Fig. 3*G*), partially in the gastrin-CCK_B_R complex (42) (Fig. 3*H*), but absent in the CCK-8-CCK_B_R complex (43). Nonetheless, they have not been systematically interpreted as a conserved and hierarchically organized receptor-side motif across species. Thus, integrating structure-guided modeling with targeted mutagenesis within a topological comparative framework across representative species provides a powerful strategy to uncover conserved functional determinants that may be missed or underemphasized by structure determination or docking alone.

### A lineage-specific modifier and a conserved sulfate-recognition core underlie subtype-selective decoding

Tyrosine sulfation is not unique to the *Aplysia* CCK system, but a conserved feature of vertebrate CCK/gastrin/caerulein signaling (33, 45, 75, 76) and also occurs in arthropod sulfakinin peptides (69, 77); moreover, disulfated endogenous CCK ligands have also been identified in several non-vertebrate species including *C. intestinalis* (78), *M. gigas* (40), *Penaeus monodon* (79), *Litopenaeus vannamei* (80) and *Homarus americanus* (81). What distinguishes the *Aplysia* CCK system is that its two receptor subtypes respond differently to sulfation number (45). This is, to our knowledge, distinct from the currently tested dual-sulfated systems of *M. gigas* and *C. intestinalis*, in which the disulfated CCK remains the most potent agonist for both receptor subtypes (40, 48).

The subtype-specific sensitivity to sulfation number provides a unique opportunity to dissect the evolutionary mechanisms underlying sulfate recognition. Our data point to residue K^7.35^ as a key contributor to this unique receptor selectivity in *Aplysia* (Figs. 6*B* and S9). The divergence between apCCKR1 and apCCKR2 does not arise from the presence or absence of K^7.35^, as both receptors retain this residue, but from its functional utilization. apCCKR1 preserved a productive K^7.35^-centered recognition mode when the second sulfate is introduced, whereas apCCKR2 appears to lose the ability to use that mode effectively (Figs. 4 and 5). A plausible mechanistic explanation, based on physicochemical properties of sulfotyrosine and receptor pocket environment, is that while sulfotyrosine enhances interaction specificity, it also introduces a strongly negatively charged and strongly hydrated group that imposes stringent electrostatic and geometric constraints (82). When two sulfations are positioned in proximity, the energetic burden of accommodation increases substantially. In this context, the apCCKR2 binding pocket appears more rigid and likely less compatible with the disulfated ligands (Fig. 5*B*), leading to a serious reconfiguration of the interaction network when both tyrosines are sulfated. By contrast, the apCCKR1 pocket exhibits greater conformational flexibility and accommodates this modification with minimal disruption (Fig. 4*B*). Future structural study is needed to validate such interpretations.

Interestingly, this lysine (K) is almost completely absent from other species. One important exception is the predicted *L. gigantea* receptors, which also retain K^7.35^ (Fig. 6*B*). Moreover, among multiple predicted *Lottia* CCK/SK-like peptides, one of them, QGAWDYDYGLGGGRFa, does contain two tyrosine residues, making dual sulfation structurally plausible (83). This suggests that *Lottia* likely employs the uncommon sulfation-selective mechanism analogous to that observed in *Aplysia*, which could be resolved in future studies. Conversely, in lineages where K^7.35^ has been replaced by hydrophobic residues (Figs. 6*B*), 7.35 appears to have undergone functional reassignment. In human CCKRs, I^7.35^ now contributes to hydrophobic packing rather than charge-based sulfate recognition (41, 42) (Fig. 7). Although structural and docking data from other species are still lacking, the evolutionary pattern strongly suggests that this hydrophobic core of 7.35 may be broadly conserved across most CCK receptors.

Beyond lineage-specific 7.35, our data suggest that CCK receptors contain an older R^ECL2 (5.26)^-E^ECL2 (5.27)^ -centered sulfate-recognition core onto which lineage- and subtype-specific solutions have been superimposed (Figs. 6*A* and S9). Specifically, sequence alignment revealed a striking boundary around reptiles/amniotes (Fig. 6*A*). In species after this boundary, positions ECL2 (5.26)-ECL2 (5.27) showed clear receptor-subtype divergence (Fig. 5*A*), accompanied by corresponding differences in receptor activation. Previous cryo-EM studies in human CCK receptors showed that for CCK_A_R, R^ECL2 (5.26)^ directly contacts sulfation (41), whereas for CCK_B_R, R^ECL2 (5.26)^ is lost and neighboring H^ECL2 (5.27)^ becomes functionally important by recognizing D^7^ (42) (Figs. 7 and S10). Combined with *Aplysia* findings, we suggest that this pattern of interaction provides explanations for the strong sulfation selectivity in CCK_A_R, but weak selectivity in CCK_B_R. A similar activation pattern in CCK receptor subtypes is present in chicken (47). Although comparable functional studies have not yet been carried out systematically in other species, we hypothesize that many representative mammals, including mouse and rat, are likely to exhibit a similar CCK_A_R/_B_R division in sulfation-dependent signaling.

By contrast, before reptiles/amniotes, this residue pair was generally conserved in both receptor subtypes, typically as R-E or R-hydrophobic combinations at position ECL2 (5.26)-ECL2 (5.27). Annelids represented the main exception, having lost the R^ECL2 (5.26)^ feature while retaining E^ECL2 (5.27)^, which possibly has taken on alternative electrostatic roles, analogous in principle to the functional reassignment observed at H^ECL2 (5.27)^ in human CCK_B_R (41, 42) (Fig. 7). This conservation implies that except for receptors carrying K^7.35^ using a more uncommon mechanism in *Aplysia* and possibly *Lottia*, these non-amniote CCK receptors are unlikely to produce subtype-divergent sulfation-selective activation, consistent with experimental evidence in several arthropod CCK/SK systems, where sulfated peptides are uniformly more potent than its nonsulfated counterparts in all subtypes (69, 84, 85).

An important implication of these findings is that sulfate recognition can arise through multiple, partially overlapping mechanisms. Variation at R^ECL2 (5.26)^ provides a widely conserved solution for sulfation-dependent activation, whereas K^7.35^ introduces an additional, less common layer that enables sensitivity to sulfation number. In *Aplysia* CCK signaling, this dual-layer architecture also explains why the two sulfations are not functionally equivalent (45). Specifically, across all modeled combinations, the residue Y^6^/sY^6^, whether sulfated or not, never participated in any ligand-receptor interactions (Figs. 4, *A*, *B*, 5, *A*, *B* and 7). By contrast, in nearly all cases, Y^8^/sY^8^ consistently interacted with K^7.35^ (Figs. 4, *A*, *B* and 5*A* and 7), and the only exception was the disulfated ligand bound to apCCKR2, in which sY^8^ shifted from K^7.35^ to R^ECL2 (5.26)^ (Figs. 5*B* and 7). This apCCKR2 pattern parallels the human CCK_A_R mechanism, in which sulfation was likewise recognized by R^ECL2 (5.26)^ (41) (Figs. 7 and S10). But in the *Aplysia* ligand-receptor complexes with apCCKR1, R^ECL2 (5.26)^ was engaged by the adjacent D^7^ rather than by sulfation (Figs. 4, *A*, *B*, 5, *A*, *B* and 7). This suggests that the apCCKR2 disulfated state may partially recapitulate a more vertebrate-like mode of sulfation recognition.

From the evolutionary perspective, these results reveal a fundamental distinction between the recognition of RFamide and sulfation. Recognition of the C-terminal RFamide is relatively conserved and organized around a stable interaction framework, as described above (Fig. 3). In contrast, sulfation recognition is evolutionarily labile and can be reassigned among different receptor residues. In *Aplysia*, sulfation recognition is primarily mediated by K^7.35^ (Figs. 4, 5 and 7), whereas in human CCK_A_R it is mediated by R^ECL2 (5.26)^, and in CCK_B_R it is largely diminished (41, 42) (Fig. 7). Thus, conserved residues do not necessarily imply conserved interaction roles; instead, receptor-side decoding can undergo selectivity-related reconfiguration to prioritize different features over evolutionary time.

This perspective also provides a predictive framework. In systems that retain R^ECL2 (5.26)^ but lack K^7.35^, such as *Drosophila*, *M. gigas*, *C. intestinalis*, and zebrafish, we propose that both subtypes would preferentially recognize sulfated ligands *via* the ECL2 mechanism, analogous to that observed in human CCK_A_R (Fig. 7). Previous docking studies did not define an equivalent R^ECL2 (5.26)^-sulfate interaction (72, 73, 74), however, re-examination using our docking framework indeed recovered this contact in models for both *Drosophila* and *Tribolium* (Fig. S11). Conversely, additional docking analyses of CCK systems in annelids *Capitella* and *Helobdella* showed that neither sulfated tyrosine nor ECL2 (5.26) participates in binding (Fig. S12), resembling the reduced sulfate-recognition mode observed in human CCK_B_R, supporting the broader applicability of our proposed sulfate-recognition model. By comparing the representative protostome *Aplysia* system with other sampled protostome and deuterostome receptors, our study provides a comparative context for interpreting CCK_A_R/_B_R divergence in humans and demonstrates that sulfation recognition can be maintained, attenuated, or reassigned among receptor-side determinants across independently diversified extant CCKR-like systems. These findings suggest that sulfation selectivity has not evolved through simple residue conservation alone, but through repeated reconfiguration of receptor-side recognition priorities in independently duplicated receptor lineages (65, 86, 87).

Collectively, this study demonstrates that co-evolution between the CCK ligands and their receptors follows a dual logic of convergence and divergence. Recognition of the C-terminal RFamide motif is hierarchically organized and strongly conserved, whereas sulfation decoding is mediated by a combination of a conserved recognition module (RE pair in ECL2) and lineage- and subtype-specific modifiers (K or other residues in TM7) that enable flexible and context-dependent selectivity. The apCCK system is therefore informative for understanding how new functional specificity can emerge while preserving shared recognition principles. More broadly, by defining peptide-feature recognition in the CCK system, this work establishes both a practical strategy for resolving neuropeptide-GPCR model ambiguity and a comparative evolutionary framework potentially applicable to other homologous neuropeptide signaling systems.

## Limitations of the study

Several limitations should be considered. First, the mechanistic interpretations relied on available cryo-EM structures of the human CCK system, whereas the apCCK system was analyzed using AI-based structural models supported by functional mutagenesis. Although this integrative approach provides strong inference, direct structural determination (*e.g.*, cryo-EM) of apCCKR-ligand complexes will be important to validate the proposed binding modes and interaction networks.

In addition, functional characterization of receptor mutants primarily emphasizes EC_50_ measurements, with limited resolution of maximal responses (E_max_). Subsequent studies should optimize the ligand concentration ranges to achieve well-defined response plateaus, enabling reliable evaluation of both potency and efficacy.

Finally, docking and mechanistic analyses were focused on mono- and disulfated apCCK1 peptides as representative ligands. A more complete understanding of ligand recognition within the CCK family will require extending these analyses to CCK2-derived peptide pairs ([sY5]-CCK2 and [sY2,sY5]-CCK2), to assess the generality of RFamide and sulfation decoding mechanisms across peptide variants.

## Materials and methods

### Molecular modeling and docking

Initial three-dimensional structures of the ligand peptides (apCCKs) were built and energy-minimized in SYBYL X-2.0 using the Amber FF99SB force field. Energy minimization was performed for a maximum of 100,000 iterations with a convergence gradient of 0.005 kcal/(mol·Å). Initial receptor topology files and coordinates were generated using the Robetta server (http://robetta.bakerlab.org/) (88). Preliminary docking trials were first carried out in Discovery Studio (89), with the putative binding region defined according to conserved GPCR active-site features. However, most output poses failed to enter the orthosteric pocket, and the few pocket-engaged models obtained were restricted to N-terminal-in orientations.

Additional docking attempts were therefore performed using AutoDock Vina (49), but most runs failed to generate valid docking outputs. Since diffdock may apply to peptide-protein docking, this platform was subsequently tested (https://huggingface.co/spaces/simonduerr/diffdock) (50). Since sulfated tyrosines in apCCKs are non-standard amino acids and diffdock requires compatible ligand input formats, ligand structures were manually built in ChemDraw and exported as SDF and mol2 files before docking (90). Nevertheless, diffdock did not yield reasonable receptor-ligand complexes. To exclude the possibility that these failures arose from receptor-model bias, receptor structures were independently rebuilt using the Swiss-Model server (https://swissmodel.expasy.org/) (91, 92), using the human CCK receptor structures deposited under PDB numbers 7EZH (41) and 7XOW (43) as templates for apCCKR1 and apCCKR2, respectively. However, none of the above modeling and docking combinations successfully produced a convincing binding mode for the apCCK signaling system.

Because of its suitability for peptide-protein docking (30), HPEPDOCK was then adopted for the final analysis (http://huanglab.phys.hust.edu.cn/hpepdock/) (51). This server supports peptide sequences containing non-standard amino acids, allowing direct submission of apCCK ligand sequences together with the pre-modeled receptor structures. Sulfated tyrosines were specified as “TYS”, whereas the C-terminal amidated phenylalanine was represented as “NFA”. No additional manual energy minimization was applied at this stage, since all docking protocols used here perform internal conformational sampling and scoring. Both Robetta-derived and Swiss-Model-derived receptor structures yielded plausible docking solutions in HPEPDOCK, although these included two mutually exclusive classes of models, namely N-terminal-in and C-terminal-in poses. For each ligand-receptor pair, blind docking was performed, and the top 10 ranked models with plausible pocket engagement were selected for interaction analysis. Recurrent interacting residues and representative poses were visualized and analyzed using PyMOL (93) and ChimeraX (94). To evaluate receptor model reliability, overall structural consistency among models was compared, and model quality was assessed using the SAVES v6.1 server (https://saves.mbi.ucla.edu/). Stereochemical properties were further evaluated by Ramachandran plot analysis with MolProbity (https://molprobity.biochem.duke.edu/) (95).

Except for apNPY, all other *Aplysia* RFamide signaling systems were modeled using the HPEPDOCK-based framework; apNPY was instead modeled by AlphaFold3 (96) and subsequently docked with HDOCK (97). CCK signaling systems in arthropods (Fig. S11) and annelids (Fig. S12) also employ the HPEPDOCK-based strategy.

### Sequence alignment

All sequence alignments were initially generated using the MUSCLE algorithm in MEGA X (98). The resulting alignments were subsequently visualized and manually inspected in BioEdit using the Graphic-View mode (https://bioedit.software.informer.com/7.2/). Predicted *Aplysia* RFamide-family receptor sequences used for cross-family comparison were obtained by searching the NCBI (https://www.ncbi.nlm.nih.gov/) and the AplysiaTools database (http://aplysiatools.org/). These latter databases include data for *Aplysia* transcriptome and genome (99). All other receptor sequences included in this study, including CCK receptors from representative invertebrate and vertebrate species, were retrieved from NCBI.

### Subjects and reagents

Experiments were performed on *A. californica* (100–350 g) obtained from Marinus, California, USA. *Aplysia* are hermaphroditic (*i.e.*, each animal has reproductive organs normally associated with both male and female sexes). Animals were maintained in circulating artificial seawater at 14 °C-16 °C and the animal room was equipped with a 24 h light cycle with the light period from 7:00 AM to 7:00 P.M. Before dissection, animals were anesthetized by injection of isotonic 333 mM MgCl_2_ (about 50% of body weight) into the body cavity. All reagents were purchased from Sigma-Aldrich unless otherwise stated. Peptides were synthesized by Sangon Biotech Pharmaceutical Co., Ltd, Guoping Pharmaceutical Co., Ltd, or Synpeptide Co., Ltd, and the purity of the peptides was >95%. Peptides were aliquoted into 50 nmol per microcentrifuge tube and stored at −20 °C until use.

### Generation of receptor plasmids

*Aplysia* CCK receptors were derived by RNA extraction and cDNA cloning. For RNA extraction, animals were anesthetized by injection of 333 mM MgCl_2_ (30–50% of body weight). Cerebral, pleural-pedal, buccal, and abdominal ganglia were then dissected and maintained in artificial seawater (460 mM NaCl, 10 mM KCl, 55 mM MgCl_2_, 11 mM CaCl_2_, 10 mM HEPES, pH 7.6) within Sylgard-lined dishes (Dow Corning). For RNA isolation, ganglia were transferred to 200 μl Trizol reagent (Sigma, T9424) and flash-frozen at −80 °C.

Frozen samples were thawed and homogenized using a plastic pestle. Trizol volume was adjusted to 1 ml, followed by 10 min incubation at room temperature. Subsequent chloroform extraction (200 μl) involved vigorous vortexing and 15 min incubation on ice. Phase separation was achieved by centrifugation (12,000×*g*, 4 °C, 15 min). The aqueous phase was combined with an equal volume of isopropanol, mixed by inversion, and precipitated at −20 °C for 2 h. RNA pellets were obtained by centrifugation (12,000×*g*, 4 °C, 15 min), washed twice with 75% ethanol, and air-dried for 5 to 10 min. Purified RNA was resuspended in 30 μl nuclease-free water, with concentration quantified *via* ND-1000 spectrophotometer (Thermo Fisher Scientific).

First-strand cDNA synthesis was performed using 1 μg total RNA with PrimeScript RT Master Mix (Takara, RR036A) under manufacturer-specified conditions. Synthesized cDNA was stored at −20 °C until subsequent PCR.

The synthesized cDNA above was used as a polymerase chain reaction (PCR) template. Specific primers were designed based on protein-coding sequences for receptors. The PCR reaction was performed with 98 °C/2 min predenaturing, 98 °C/10 s denaturing, ∼64 °C/15 s annealing, 72 °C/30 s extension, and 72 °C/5 min re-extension for 35 cycles. The PCR products were subcloned into vector pcDNA3.1(+) or pcDNA3.1(+)-FLAG (for ELISA experiments only, see “Cell surface expression analysis by ELISA” section) and sequenced to ensure the sequences were correct.

### Mutagenesis of the apCCKRs

In the first set of experiments, mutagenesis of the receptors was performed with a FLAG tag. Specifically, construction of the mutants was performed employing the full-length receptor cDNA cloned into the pcDNA3.1(+) plasmid. Site-directed mutagenesis was performed using the site-directed mutagenesis kit (Sangon Biotech), following the manufacturer’s instructions. Briefly, forward and reverse primers containing the expected mutation were mixed with kit components, and 10 ng of pcDNA3.1(+)-receptors was used as the mutation template. After 14 to 18 rounds of PCR amplification, 1 μl of DpnI was added and incubated at 37 °C for 1 h to digest the template. Mutants were confirmed *via* DNA sequencing. IP1 accumulation assay with apCCKRs and mutants with FLAG tags was performed using procedures described in “IP1 accumulation assay” section.

### Cell culture and transfection

The CHO-K1 cells (Procell, CL-0062) were cultured in F-12K medium (Gibco, 21127-022) with 10% fetal bovine serum (FBS) (Genial, G11-70500) at 37 °C in a humidified incubator containing 5% CO_2_. Cell culture dishes (Corning, 430166) were used for culture until cells reached the logarithmic growth phase. Then, the medium was discarded, and cells were washed twice with PBS and digested with trypsin. Transfection experiments were performed when the cells were grown to 70 to 90% confluence and using jetPRIME (Polyplus Transfection, PT-114-15) transfection reagent, according to the manufacturer’s instructions. Specifically, in all experiments in this study, for each 35 mm dish, 2 μg of the putative receptor plasmids [in pcDNA3.1(+) or pcDNA3.1(+)-FLAG] and 2 μg Gαq plasmids [in pcDNA3.1(+)] were mixed with 200 μl jetPRIME Buffer (Lot# 0000000396), followed by the addition of 6 μl jetPRIME.

The promiscuous Gαq construct is an engineered G protein with broad receptor compatibility that routes signaling from a wide range of GPCRs through the IP3 signaling pathway (56). This strategy provides a common IP1-based functional readout regardless of the receptor’s endogenous G-protein coupling preferences (see “IP1 accumulation assay section”). The expression of this promiscuous Gαq protein was not independently quantified in the present study, although Gαq plasmids were transfected for all wild-type and mutant receptors at the same concentration and duration under identical transfection conditions, and therefore Gαq proteins presumably are expressed at similar levels with all receptors.

The CHO cells with the reagents added above were mixed gently and incubated at room temperature for 15 min. The DNA/jetPRIME mixture dropwise was then added to the dish, and the cells were incubated at 37 °C in 5% CO_2_ overnight.

### IP1 accumulation assay

Receptor activation was quantified using an inositol monophosphate (IP1) accumulation assay, which measures IP1, generated from hydrolysis of the second messenger, inositol triphosphate (IP3), downstream of the Gαq-mediated signaling (56, 100, 101, 102, 103, 104, 105). Following ligand stimulation of transfected CHO-K1 cells, IP3 produced *via* phospholipase C activity is rapidly metabolized to IP1, which accumulates and serves as a stable quantitative proxy for receptor activation. As described above, all wild-type and mutant receptors were co-transfected with the promiscuous Gαq construct to provide a common IP1-based functional readout (56).

On the third day, transfected cells were trypsinized and reseeded in 384-well tissue culture-treated plates (Corning, 3570) at a density of 20,000 cells/well in F-12K and 10% FBS at 37 °C in 5% CO_2_ overnight. The next day, the medium in wells was discarded and exposed to potential peptide agonists in 14 μl stimulation buffer (Lot No. 621P1FDC) for 1h. Peptide agonists, ranging in concentrations from 10^−12^ to 10^−4^ M, were used to construct dose-response curves. The activation of the putative receptors was detected by monitoring intracellular IP1 concentration using an IP-One Gq kit (Cisbio, 62IPAPEB) in Tecan Spark following the manufacturer’s instructions with minor modifications (for using 0.5 × reagents for the anti-IP1-cryptate and IP1-d2 reagents). Specifically, after adding the detection solution to each well, the sealed plate was incubated for a minimum of 1h at RT protected from light, and each plate was then read with Spark (TECAN, USA) with an HTRF compatible setup: excitation at 320 nm and emitted light at 620 and 665 nm.

### Cell surface expression analysis by ELISA

Cell surface expression determination of apCCKR and mutant receptors, all with FLAG tags, was performed using a procedure modified from previous work (14, 31, 106, 107, 108). Specifically, CHO-K1 cells were maintained in F-12K medium with 10% FBS at 37 °C in 5% CO_2_. When the cells were grown to 70 to 90% confluence, transfection experiments were performed. Briefly, in each well, 2 μg of the receptor plasmids [in pcDNA3.1(+)-FLAG] were mixed with 200 μl of jetPRIME buffer, followed by the addition of 6 μl of jetPRIME and incubated at room temperature for 15 min. The DNA/jetPRIME mixture dropwise was then added to the dish. The cells were incubated at 37 °C in 5% CO_2_ overnight. After 24 h, transfected cells were plated in 96-well white clear-bottom cell culture plates (Corning, 3610) at a density of 20,000 cells in 100 μl per well and incubated overnight. The following day, culture media was aspirated and cells were washed twice with 200 μl of 1 × PBS (Procell, PB180327). Then, 100 μl of 1 × PBS containing 5% (w/v) bovine serum albumin was added to each well and incubated at room temperature. After 30 min, 100 μl of 1:10,000 anti-FLAG M2-HRP conjugate (Sigma-Aldrich, Cat A8592) was added to each well and incubated for 30 min at 37 °C. Cells were washed twice with 200 μl of 1 × PBS and then incubated with 200 μl of TMB Chromogen Solution (Sangon Biotech, E661007) for 30 min at 37 °C in the dark. Finally, 50 μl of ELISA Stopping Solution (Sangon Biotech, E661006) was added to each well to stop the reaction. The absorbance at 450 nm was measured using a Tecan Spark microplate reader. In each experiment, expression of the mutant was assessed and compared with that measured for the corresponding wildtype receptor in the same experiment. All data are summarized in Table S2.

### Statistical analysis

Dose-response curves and bar graphs for experimental data were plotted using Prism software (GraphPad, https://www.graphpad.com/). Data are presented as mean ± SD of at least three independent experiments. Statistical tests were performed using Prism software. Comparisons between two groups were performed using a two-tailed unpaired Student’s *t* test, whereas comparisons among multiple groups were performed using one-way ANOVA followed by Tukey's *post hoc* tests. Statistical significance was set at *p* < 0.05.

## Data availability

The original contributions presented in the study are included in the article and Supplementary information.

## Supporting information

This article contains supporting information , which includes Table S1–S4 and Figures S1–S12 (17, 41, 42, 43, 72, 73, 74, 109).

## Conflict of interest

The authors declare that they have no conflicts of interest with the contents of this article.

## References

[1] Abid M.S.R., Mousavi S., Checco J.W. (2021). Identifying receptors for neuropeptides and peptide hormones: challenges and recent progress. ACS Chem. Biol..

[2] Mafi A., Kim S.-K., Goddard III W.A. (2022). The mechanism for ligand activation of the GPCR–G protein complex. Proc. Natl. Acad. Sci. U. S. A..

[3] Zhang M., Chen T., Lu X., Lan X., Chen Z., Lu S. (2024). G protein-coupled receptors (GPCRs): advances in structures, mechanisms and drug discovery. Signal. Transd. Target. Therapy.

[4] Hauser A.S., Attwood M.M., Rask-Andersen M., Schiöth H.B., Gloriam D.E. (2017). Trends in GPCR drug discovery: new agents, targets and indications. Nat. Rev. Drug Discov..

[5] Sriram K., Insel P.A. (2018). G protein-coupled receptors as targets for approved drugs: how many targets and how many drugs?. Mol. Pharmacol..

[6] Yang D., Zhou Q., Labroska V., Qin S., Darbalaei S., Wu Y. (2021). G protein-coupled receptors: structure-and function-based drug discovery. Signal Transd. Target. Ther..

[7] Lorente J.S., Sokolov A.V., Ferguson G., Schiöth H.B., Hauser A.S., Gloriam D.E. (2025). GPCR drug discovery: new agents, targets and indications. Nat. Rev. Drug Discov..

[8] Conflitti P., Lyman E., Sansom M.S., Hildebrand P.W., Gutiérrez-de-Terán H., Carloni P. (2025). Functional dynamics of G protein-coupled receptors reveal new routes for drug discovery. Nat. Rev. Drug Discov..

[9] Craig A.G., Bandyopadhyay P., Olivera B.M. (1999). Post-translationally modified neuropeptides from *Conus* venoms. Eur. J. Biochem..

[10] Larhammar D., Sundström G., Dreborg S., Daza D.O., Larsson T.A. (2009). Major genomic events and their consequences for vertebrate evolution and endocrinology. Ann. New York Acad. Sci..

[11] Saikia S., Bordoloi M. (2019). Molecular docking: challenges, advances and its use in drug discovery perspective. Curr. Drug Targets.

[12] Patwardhan A., Cheng N., Trejo J. (2021). Post-translational modifications of g protein–coupled receptors control cellular signaling dynamics in space and time. Pharmacol. Rev..

[13] Shonberg J., Kling R.C., Gmeiner P., Löber S. (2015). GPCR crystal structures: medicinal chemistry in the pocket. Bioorg. Med. Chem..

[14] Zhuang Y., Xu P., Mao C., Wang L., Krumm B., Zhou X.E. (2021). Structural insights into the human D1 and D2 dopamine receptor signaling complexes. Cell.

[15] You C., Zhang Y., Xu P., Huang S., Yin W., Eric Xu H. (2022). Structural insights into the peptide selectivity and activation of human neuromedin U receptors. Nat. Commun..

[16] Zhao W., Zhang W., Wang M., Lu M., Chen S., Tang T. (2022). Ligand recognition and activation of neuromedin U receptor 2. Nat. Commun..

[17] Park C., Kim J., Ko S.-B., Choi Y.K., Jeong H., Woo H. (2022). Structural basis of neuropeptide Y signaling through Y1 receptor. Nat. Commun..

[18] Yang F., Zhang H., Meng X., Li Y., Zhou Y., Ling S. (2022). Structural insights into thyrotropin-releasing hormone receptor activation by an endogenous peptide agonist or its orally administered analogue. Cell Res..

[19] Wiggenhorn A.L., Abuzaid H.Z., Coassolo L., Li V.L., Tanzo J.T., Wei W. (2023). A class of secreted mammalian peptides with potential to expand cell-cell communication. Nat. Commun..

[20] Wen X., Shang P., Chen H., Guo L., Rong N., Jiang X. (2025). Evolutionary study and structural basis of proton sensing by *Mus* GPR4 and *Xenopus* GPR4. Cell.

[21] Wang J.-L., Sha X.-Y., Shao Y., Zhang Z.-H., Huang S.-M., Lin H. (2026). Elucidating pathway-selective biased CCK_B_R agonism for Alzheimer’s disease treatment. Cell.

[22] Han X., Zhang M.-H., Rong N.-K., Zhu K.-K., Pei Y., Ge X.-Y. (2026). Mechanistic insights into fatty acid odor detection mediated by class II olfactory receptors. Cell.

[23] Tobi D., Bahar I. (2005). Structural changes involved in protein binding correlate with intrinsic motions of proteins in the unbound state. Proc. Natl. Acad. Sci. U. S. A..

[24] Latorraca N.R., Venkatakrishnan A., Dror R.O. (2017). GPCR dynamics: structures in motion. Chem. Rev..

[25] Shihoya W., Iwama A., Sano F.K., Nureki O. (2024). Cryo-EM advances in GPCR structure determination. J. Biochem..

[26] Vilar S., Cozza G., Moro S. (2008). Medicinal chemistry and the molecular operating environment (MOE): application of QSAR and molecular docking to drug discovery. Curr. Top. Med. Chem..

[27] Beuming T., Sherman W. (2012). Current assessment of docking into GPCR crystal structures and homology models: successes, challenges, and guidelines. J. Chem. Inf. Model..

[28] Ciemny M., Kurcinski M., Kamel K., Kolinski A., Alam N., Schueler-Furman O. (2018). Protein–peptide docking: opportunities and challenges. Drug Discov. Today.

[29] Torres P.H., Sodero A.C., Jofily P., Silva-Jr F.P. (2019). Key topics in molecular docking for drug design. Int. J. Mol. Sci..

[30] Weng G., Gao J., Wang Z., Wang E., Hu X., Yao X. (2020). Comprehensive evaluation of fourteen docking programs on protein–peptide complexes. J. Chem. Theor. Comput..

[31] Guo S.-Q., Li Y.-D., Chen P., Zhang G., Wang H.-Y., Jiang H.-M. (2022). AI protein structure prediction-based modeling and mutagenesis of a protostome receptor and peptide ligands reveal key residues for their interaction. J. Biol. Chem..

[32] Price D.A., Greenberg M.J. (1977). Structure of a molluscan cardioexcitatory neuropeptide. Science.

[33] Miller L.J., Jardine I., Weissman E., Go V.L.W., Speicher D. (1984). Characterization of cholecystokinin from the human brain. J. Neurochem..

[34] Nachman R.J., Holman G.M., Haddon W.F., Ling N. (1986). Leucosulfakinin, a sulfated insect neuropeptide with homology to gastrin and cholecystokinin. Science.

[35] Takahashi T., Kobayakawa Y., Muneoka Y., Fujisawa Y., Mohri S., Hatta M. (2003). Identification of a new member of the GLWamide peptide family: physiological activity and cellular localization in cnidarian polyps. Comp. Biochem. Physiol. B: Biochem. Mol. Biol..

[36] Walker R.J., Papaioannou S., Holden-Dye L. (2009). A review of FMRFamide-and RFamide-like peptides in metazoa. Invert. Neurosci..

[37] Baldwin G.S., Patel O., Shulkes A. (2010). Evolution of gastrointestinal hormones: the cholecystokinin/gastrin family. Curr. Opin. Endocrinol. Diabetes Obes..

[38] Elphick M.R., Mirabeau O. (2014). The evolution and variety of RFamide-type neuropeptides: insights from deuterostomian invertebrates. Front. Endocrinol..

[39] Osugi T., Son Y.L., Ubuka T., Satake H., Tsutsui K. (2016). RFamide peptides in agnathans and basal chordates. Gen. Comp. Endocrinol..

[40] Schwartz J., Dubos M.-P., Pasquier J., Zatylny-Gaudin C., Favrel P. (2018). Emergence of a cholecystokinin/sulfakinin signalling system in Lophotrochozoa. Sci. Rep..

[41] Liu Q., Yang D., Zhuang Y., Croll T.I., Cai X., Dai A. (2021). Ligand recognition and G-protein coupling selectivity of cholecystokinin A receptor. Nat. Chem. Biol..

[42] Zhang X., He C., Wang M., Zhou Q., Yang D., Zhu Y. (2021). Structures of the human cholecystokinin receptors bound to agonists and antagonists. Nat. Chem. Biol..

[43] Ding Y., Zhang H., Liao Y.-Y., Chen L.-N., Ji S.-Y., Qin J. (2022). Structural insights into human brain–gut peptide cholecystokinin receptors. Cell Discov..

[44] Jin S., Guo S., Xu Y., Li X., Wu C., He X. (2024). Structural basis for recognition of 26RFa by the pyroglutamylated RFamide peptide receptor. Cell Discov..

[45] Zhang G., Ding X.-Y., Romanova E.V., Liu C.-P., Barry M.A., Doda A. (2025). Synaptic and intrinsic plasticity mediated by CCK-type signaling coordinates behavioral changes during motivational state shifts. Cell Rep..

[46] Wu Z., Chen G., Qiu C., Yan X., Xu L., Jiang S. (2024). Structural basis for the ligand recognition and G protein subtype selectivity of kisspeptin receptor. Sci. Adv..

[47] Wan Y., Deng Q., Zhou Z., Deng Y., Zhang J., Li J. (2023). Cholecystokinin (CCK) and its receptors (CCK1R and CCK2R) in chickens: functional analysis and tissue expression. Poult. Sci..

[48] Sekiguchi T., Ogasawara M., Satake H. (2012). Molecular and functional characterization of cionin receptors in the ascidian, *Ciona intestinalis*: the evolutionary origin of the vertebrate cholecystokinin/gastrin family. J. Endocrinol..

[49] Morris G.M., Huey R., Olson A.J. (2008). Using autodock for ligand-receptor docking. Curr. Protoc. Bioinform..

[50] Corso G., Stärk H., Jing B., Barzilay R., Jaakkola T. (2022). Diffdock: diffusion steps, twists, and turns for molecular docking. arXiv.

[51] Zhou P., Jin B., Li H., Huang S.-Y. (2018). HPEPDOCK: a web server for blind peptide–protein docking based on a hierarchical algorithm. Nucleic Acids Res..

[52] Bortolato A., Doré A.S., Hollenstein K., Tehan B.G., Mason J.S., Marshall F.H. (2014). Structure of Class B GPCRs: new horizons for drug discovery. Br. J. Pharmacol..

[53] Liu H., Sun D., Myasnikov A., Damian M., Baneres J.-L., Sun J. (2021). Structural basis of human ghrelin receptor signaling by ghrelin and the synthetic agonist ibutamoren. Nat. Commun..

[54] Jiang W., Zheng S. (2022). Structural insights into galanin receptor signaling. Proc. Natl. Acad. Sci. U. S. A..

[55] Isberg V., De Graaf C., Bortolato A., Cherezov V., Katritch V., Marshall F.H. (2015). Generic GPCR residue numbers–aligning topology maps while minding the gaps. Trends Pharmacol. Sci..

[56] Sharma S., Checco J.W. (2021). Methods in Cell Biology.

[57] Taussig R., Scheller R.H. (1986). The *Aplysia* FMRFamide gene encodes sequences related to mammalian brain peptides. DNA and Cell Biology.

[58] Rajpara S.M., Garcia P.D., Roberts R., Eliassen J.C., Owens D.F., Maltby D. (1992). Identification and molecular cloning of a neuropeptide Y homolog that produces prolonged inhibition in *Aplysia* neurons. Neuron.

[59] Aloyz R.S., DesGroseillers L. (1995). Processing of the L5–67 precursor peptide and characterization of LUQIN in the LUQ neurons of *Aplysia californica*. Peptides.

[60] Cropper E.C., Brezina V., Vilim F.S., Harish O., Price D.A., Rosen S. (1994). FRF peptides in the ARC neuromuscular system of *Aplysia*: purification and physiological actions. J. Neurophysiol..

[61] Wang H.-Y. (2023).

[62] Janssen T., Meelkop E., Lindemans M., Verstraelen K., Husson S.J., Temmerman L. (2008). Discovery of a cholecystokinin-gastrin-like signaling system in nematodes. Endocrinology.

[63] Tikhonova I.G., Gigoux V., Fourmy D. (2019). Understanding peptide binding in class AG protein-coupled receptors. Mol. Pharmacol..

[64] Jékely G. (2013). Global view of the evolution and diversity of metazoan neuropeptide signaling. Proc. Natl. Acad. Sci. U. S. A..

[65] Mirabeau O., Joly J.-S. (2013). Molecular evolution of peptidergic signaling systems in bilaterians. Proc. Natl. Acad. Sci. U. S. A..

[66] Wang H.-Y., Yu K., Liu W.-J., Jiang H.-M., Guo S.-Q., Xu J.-P. (2023). Molecular characterization of two Wamide neuropeptide signaling systems in mollusk *Aplysia*. ACS Chem. Neurosci..

[67] Brown M.R., Crim J.W., Arata R.C., Cai H.N., Chun C., Shen P. (1999). Identification of a *Drosophila* brain-gut peptide related to the neuropeptide Y family. Peptides.

[68] Yu N., Smagghe G. (2014). CCK (-like) and receptors: structure and phylogeny in a comparative perspective. Gen. Comp. Endocrinol..

[69] Nässel D.R., Wu S.-F. (2022). Cholecystokinin/sulfakinin peptide signaling: conserved roles at the intersection between feeding, mating and aggression. Cell Mol. Life Sci..

[70] Fields L., Dang T.C., Tran V.N.H., Ibarra A.E., Li L. (2025). Decoding neuropeptide complexity: advancing neurobiological insights from invertebrates to vertebrates through evolutionary perspectives. ACS Chem. Neurosci..

[71] Yang H., Fratta W., Majane E., Costa E. (1985). Isolation, sequencing, synthesis, and pharmacological characterization of two brain neuropeptides that modulate the action of morphine. Proc. Natl. Acad. Sci. U. S. A..

[72] Leander M., Heimonen J., Brocke T., Rasmussen M., Bass C., Palmer G. (2016). The 5-amino acid N-terminal extension of non-sulfated drosulfakinin II is a unique target to generate novel agonists. Peptides.

[73] Nichols R., Bass C., Katanski C. (2022). Structure-activity relationship data and ligand-receptor interactions identify novel agonists consistent with sulfakinin tissue-specific signaling in *Drosophila melanogaster* heart. Front. Biosci. Landmark.

[74] Yu N., Zotti M.J., Scheys F., Braz A.S., Penna P.H., Nachman R.J. (2015). Flexibility and extracellular opening determine the interaction between ligands and insect sulfakinin receptors. Sci. Rep..

[75] Corbeil D., Huttner W.B., Lennarz W.J., Lane M.D. (2013). Encyclopedia of Biological Chemistry.

[76] Roelants K., Fry B.G., Norman J.A., Clynen E., Schoofs L., Bossuyt F. (2010). Identical skin toxins by convergent molecular adaptation in frogs. Curr. Biol..

[77] Nachman R.J., Holman G.M., Haddon W.F. (1988). Structural aspects of gastrin/CCK-like insect leucosulfakinins and FMRF-amide. Peptides.

[78] Johnsen A.H., Rehfeld J. (1990). Cionin: a disulfotyrosyl hybrid of cholecystokinin and gastrin from the neural ganglion of the protochordate *Ciona intestinalis*. J. Biol. Chem..

[79] Johnsen A.H., Duve H., Davey M., Hall M., Thorpe A. (2000). Sulfakinin neuropeptides in a crustacean: isolation, identification and tissue localization in the tiger prawn *Penaeus monodon*. Eur. J. Biochem..

[80] Torfs P., Baggerman G., Meeusen T., Nieto J., Nachman R., Calderon J. (2002). Isolation, identification, and synthesis of a disulfated sulfakinin from the central nervous system of an arthropods the white shrimp *Litopenaeus vannamei*. Biochem. Biophys. Res. Commun..

[81] Dickinson P.S., Stevens J.S., Rus S., Brennan H.R., Goiney C.C., Smith C.M. (2007). Identification and cardiotropic actions of sulfakinin peptides in the American lobster *Homarus americanus*. J. Exp. Biol..

[82] Stewart V., Ronald P.C. (2022). Sulfotyrosine residues: interaction specificity determinants for extracellular protein–protein interactions. J. Biol. Chem..

[83] Zatylny-Gaudin C., Favrel P. (2014). Diversity of the RFamide peptide family in mollusks. Front. Endocrinol..

[84] Zels S., Verlinden H., Dillen S., Vleugels R., Nachman R.J., Broeck J.V. (2014). Signaling properties and pharmacological analysis of two sulfakinin receptors from the red flour beetle, *Tribolium castaneum*. Plos One.

[85] Bloom M., Lange A.B., Orchard I. (2019). Identification, functional characterization, and pharmacological analysis of two sulfakinin receptors in the medically-important insect *Rhodnius prolixus*. Sci. Rep..

[86] Yun S., Furlong M., Sim M., Cho M., Park S., Cho E.B. (2015). Prevertebrate local gene duplication facilitated expansion of the neuropeptide GPCR superfamily. Mol. Biol. Evol..

[87] Elphick M.R., Mirabeau O., Larhammar D. (2018). Evolution of neuropeptide signalling systems. J. Exp. Biol..

[88] Kim D.E., Chivian D., Baker D. (2004). Protein structure prediction and analysis using the Robetta server. Nucleic Acids Res..

[89] Pawar S.S., Rohane S.H. (2021). Review on discovery studio: an important tool for molecular docking. Asian J. Res. Chem..

[90] Mendelsohn L.D. (2004). ChemDraw 8 ultra, windows and macintosh versions. J. Chem. Inf. Computer Sci..

[91] Schwede T., Kopp J., Guex N., Peitsch M.C. (2003). SWISS-MODEL: an automated protein homology-modeling server. Nucleic Acids Res..

[92] Waterhouse A., Bertoni M., Bienert S., Studer G., Tauriello G., Gumienny R. (2018). SWISS-MODEL: homology modelling of protein structures and complexes. Nucleic Acids Res..

[93] DeLano W.L. (2002). PyMOL: an open-source molecular graphics tool. CCP4 Newsl. Protein Crystallogr..

[94] Pettersen E.F., Goddard T.D., Huang C.C., Meng E.C., Couch G.S., Croll T.I. (2021). UCSF ChimeraX: structure visualization for researchers, educators, and developers. Protein Sci..

[95] Chen V.B., Arendall W.B., Headd J.J., Keedy D.A., Immormino R.M., Kapral G.J. (2010). MolProbity: all-atom structure validation for macromolecular crystallography. Biol. Crystallogr..

[96] Jumper J., Evans R., Pritzel A., Green T., Figurnov M., Ronneberger O. (2021). Highly accurate protein structure prediction with AlphaFold. Nature.

[97] Yan Y., Tao H., He J., Huang S.-Y. (2020). The HDOCK server for integrated protein–protein docking. Nat. Protoc..

[98] Kumar S., Stecher G., Li M., Knyaz C., Tamura K. (2018). Mega X: molecular evolutionary genetics analysis across computing platforms. Mol. Biol. Evol..

[99] Orvis J., Albertin C.B., Shrestha P., Chen S., Zheng M., Rodriguez C.J. (2022). The evolution of synaptic and cognitive capacity: insights from the nervous system transcriptome of *Aplysia*. Proc. Natl. Acad. Sci. U. S. A..

[100] Bauknecht P., Jékely G. (2015). Large-scale combinatorial deorphanization of *Platynereis* neuropeptide GPCRs. Cell Rep..

[101] Jiang H.-M., Yang Z., Xue Y.-Y., Wang H.-Y., Guo S.-Q., Xu J.-P. (2022). Identification of an allatostatin C signaling system in mollusc *Aplysia*. Sci. Rep..

[102] Xu J.-P., Ding X.-Y., Guo S.-Q., Wang H.-Y., Liu W.-J., Jiang H.-M. (2023). Characterization of an *Aplysia* vasotocin signaling system and actions of posttranslational modifications and individual residues of the ligand on receptor activity. Front. Pharmacol..

[103] Fu P., Mei Y.-S., Liu W.J., Chen P., Jin Q.-C., Guo S.Q. (2023). Identification of three elevenin receptors and roles of elevenin disulfide bond and residues in receptor activation in *Aplysia* californica. Sci. Rep..

[104] Xu J.-P., Ding X.-Y., Jin Q.-C., Zhang Y.-L., Chang J.-H., Jiang H.-M. (2025). Identification of a G protein-coupled receptor for buccalin-type peptides in the mollusk *Aplysia*: evolutionary insights into neuropeptide signaling. ACS Omega.

[105] Liu C.-P., Fu P., Pang D., McManus J.M., Romanova E.V., Thompson C. (2026). A brain-gut excitatory peptide/CCHamide homolog regulates satiation and motivational state transitions in the *Aplysia* feeding circuit. J. Biol. Chem..

[106] Lourenço E.V., Roque-Barreira M.-C. (2009). Immunocytochemical Methods and Protocols.

[107] Newton C.L., Anderson R.C., Katz A.A., Millar R.P. (2016). Loss-of-function mutations in the human luteinizing hormone receptor predominantly cause intracellular retention. Endocrinology.

[108] Mao R.-T., Guo S.-Q., Zhang G., Li Y.-D., Xu J.-P., Wang H.-Y. (2024). Two C-terminal isoforms of *Aplysia* tachykinin–related peptide receptors exhibit phosphorylation-dependent and phosphorylation-independent desensitization mechanisms. J. Biol. Chem..

[109] Iwama A., Kise R., Akasaka H., Sano F.K., Oshima H.S., Inoue A. (2024). Structure and dynamics of the pyroglutamylated RF-amide peptide QRFP receptor GPR103. Nat. Commun..

