# Supplementary material for: Decoding of amidated aromatic C-terminus and sulfation by cholecystokinin receptors reveals conserved and divergent evolutionary mechanisms
Source: J Biol Chem. 2026 Jul 13;302(9):113327. doi: 10.1016/j.jbc.2026.113327 (PMC13485628; doi:10.1016/j.jbc.2026.113327)
Supplement: Supplementary file 1 [file mmc1.docx]

**Supplementary Information**

**Decoding of amidated aromatic C-terminus and sulfation by cholecystokinin receptors reveals conserved and divergent evolutionary mechanisms**

Jian-Hui Chang^1,#^, Shao-Qian Wu^1,#^, Xue-Ying Ding^1^, Xing Pan^1^, Hui-Ying Wang^1^, Yu-Xuan He^1^, Lin Xi^1^, Yu-Shuo Mei^1^, Cheng-Yi Liu^1^, Cui-Ping Liu^1^, Rui-Ting Mao^1^, Fan Li^1^, Yi-Long Zhang^1^, Jiang-Yue Li^1^, Guo Zhang^1,2,*^, Jian Jing^1,*^

*^1^State Key Laboratory of Pharmaceutical Biotechnology, Department of Neurology and Medical Psychology, Nanjing Drum Tower Hospital, The affiliated Hospital of Nanjing University Medical School, Institute for Brain Sciences, School of Life Sciences, Nanjing University, Nanjing 210023, Jiangsu, China.*

*^2^Department of Pathophysiology, School of Basic Medical Sciences, Institute of Metabolism and Health, Henan University, Kaifeng, Henan 475004, China.*

^#^ These authors contributed equally to this work.

* For correspondence: Jian Jing (jingj01@live.com), Guo Zhang (zhangguonanda@163.com).

There are twelve supporting figures **(Figs. S1-S12)** and three supporting tables **(Tables S2-S4)** included in this document. Table S1 is a separated excel file.

**Figure S1.** Overall structures of apCCKR1 predicted by Swiss-Model and Robetta. **A,** Orthogonal views of two apCCKR1 receptors: light green (Swiss model) and red (Robetta). **B,** Structural comparison of the two models. ECL1-3 are shown in the box for comparison. **C,** Comparison of the seven TM regions from both models. ECL, Extracellular loop; TM, transmembrane domain.

**Figure S2.** Overall structures of apCCKR2 predicted by Swiss-Model and Robetta. **A,** Orthogonal views of two apCCKR2 receptors: light green (Swiss model) and purple (Robetta). **B,** Structural comparison of the two models. ECL1-3 are shown in the box for comparison. **C,** Comparison of the seven TM regions from both models.

**Figure S3. Validation and quality assessment of apCCKR1 and apCCKR2 predicted models. A-B,** Ramachandran plots of apCCKR1 and apCCKR2 models generated by the Robetta server, with the favored and allowed regions colored in light and dark blue, respectively. Z-scores and ERRAT-derived non-bonded interaction scores are shown. **C-D,** Evaluation index for models generated by Swiss-Model. Across all four models, over 90% of residues fall within the favored region, while Z-scores are mostly less than 2, and ERRAT scores exceed 90, supporting high structural reliability.

**Figure S4. Cutting-side views of human CCK-8 in ligand-receptor complexes resolved by Cryo-EM.** The human CCK8-CC_A_R complex is rendered from experimentally resolved structural data (PDB: 7XOU), while the CCK8-CC_B_R complex is redrawn based on models from (PDB: 7XOX) (1). In both cases, the receptor was sectioned from the front toward the center of the transmembrane bundle until the ligand was fully visible within the orthosteric pocket. This cutaway representation was used to directly compare peptide orientation in the two human receptor subtypes. In both complexes, **CCK-8 adopts a conserved C-terminal-in binding mode**, with the **N-terminus exposed toward the extracellular side** and the **C-terminus deeply inserted into the receptor-binding pocket**. These human receptor structures, therefore provided an important structural reference for evaluating the alternative **N-terminal-in** and **C-terminal-in** docking poses generated for the apCCK system. The approximate positions of residues associated with evolutionary divergence and convergence in CCK receptors are circled in the figure.

**Figure S5. Functional validation of RFamide-recognition residues in apCCKR1 and apCCKR2 using two additional endogenous apCCK peptides. A, B,** Representative dose-response curves showing activation of apCCKR1 and its Y371A, N344A and R125A mutants by [sY8]-CCK1 and [sY5]-CCK2. All curves were normalized to 100% of the maximal response induced by the corresponding peptide in wild-type apCCKR1. Bar graphs summarizing the pEC50 values for [sY8]-CCK1 and [sY5]-CCK2 at wild-type apCCKR1 and the two mutants. For panel **A**, n = 3. One-way ANOVA, F (2, 6) = 84.25, P < 0.0001. For panel **B**, n ≥ 3. One-way ANOVA, F (3, 9) = 19.27, P = 0.0003. **C, D,** Representative dose-response curves showing activation of apCCKR2 and its Y465A, S438A and R201A mutants by [sY8]-CCK1 and [sY5]-CCK2. All curves were normalized to 100% of the maximal response induced by the corresponding peptide in wild-type apCCKR2. Bar graphs summarizing the pEC_50_ values for [sY8]-CCK1 and [sY5]-CCK2 at wild-type apCCKR2 and the two mutants. For panel **C**, n = 3. Paired two-tailed t test: ND, not detected. For panel **D**, n = 3. ND, not detected. One-way ANOVA, F (3, 8) = 28.22, P = 0.0001. Tukey’s post hoc test: n.s., not significant; * P < 0.05; ** P < 0.01; *** P < 0.001; **** P < 0.0001. Error bars indicate SD. The activation curves and data of the R125A and R201A mutants have been presented in **Fig. 2**. These data show that the functional importance of the identified RFamide-recognition residues is not restricted to the representative ligand [sY6,sY8]-CCK1, but is also retained for the additional endogenous peptides [sY8]-CCK1 and [sY5]-CCK2, supporting a broader role of these residues in CCK/RFamide recognition. p[EC_50_], -log_10_(EC_50_).

 (Page. 1)

**Figure S6. Sequence alignment of predicted invertebrate (*Aplysia*) RFamide-family receptors and vertebrate (human) RFamide-like (RFamide, DFamide, RYamide) receptors. A,** Amino acid sequences of representative RFamide-family receptors from *Aplysia* (predicted from the databases) and human were aligned using BioEdit with the MUSCLE multiple alignment algorithm (graphic view). The alignment includes the predicted *Aplysia* RFamide receptors, which were used for the subsequent preliminary molecular modeling analyses. Predicted TM domains, identified by DeepTMHMM, are indicated by black lines. Conserved Class A GPCR motifs in ICL2 (D/ERY) and TM7 (NPxxY/HxxY) are marked in the alignment. Residues at 3.29, 6.55, and 7.43 positions are labeled in this figure. Detailed information on these sequences is provided in **Table S3.** NPYR1, neuropeptide Y receptor 1; QRFPR, pyroglutamylated RFamide peptide receptor; PrRPR, prolactin-releasing peptide receptor; NPFFR1/2, neuropeptide FF receptor 1/2; KissR, kisspeptin receptor; FRFaR, FRFamide receptor; LuqinR, luqin receptor; FMRFaR, FMRFamide receptor.

**Figure S7.** Expanded alignment highlighting the three representative receptor-side positions discussed in this study, based on **Figure S6**, shown separately for easier comparison across receptors. These sites illustrate different levels of conservation in peptide C-terminal recognition: 7.43 (***) represents the most deeply conserved anchoring position, 6.55 (**) indicates a CCK-biased layer that remains relatively conserved within the CCK system, and 3.29 (*) marks the most variable position, consistent with its role as a more plastic tuning site. # indicates that the structure has been resolved and that position 7.43 has been detected to contact the receptor. # denotes receptors that have been resolved by cryo-EM or have undergone functional experiments.

**Figure S8. Preliminary docking analysis of predicted *Aplysia* RFamide-family receptors with their corresponding peptide ligands.** Each model is shown as an overall receptor structure together with a zoomed-in view of the boxed region, although the overall and enlarged views are not always presented from the same viewing angle. All predicted *Aplysia* RFamide-family receptor sequences shown in **Figure S8** and listed in **Table S3** were subjected to molecular docking with their corresponding peptide ligands, followed by preliminary interaction analysis. **A,** In the apLuqin-apLuqinR model, peptide residue F^10^ forms a hydrophobic interaction with receptor residue M341^7.43^. **B,** In the apFRFa-A-apFRFaR model, the peptide is predicted to interact with receptor residue Y371^7.43^ through a hydrogen bond. **C,** In the apFMRFa-apFMRFaR model, peptide residue F forms a hydrogen bond with receptor residue M340^7.43^. **D,** In the apNPY-apNPYR model, receptor residue M307^7.43^ does not show a clearly assigned interaction with peptide residue F^40^ in the docking output. However, the two residues are positioned in close spatial proximity, suggesting that a hydrophobic interaction is still highly likely. This interpretation is also consistent with experimentally resolved human NPY receptor structures (2, 3), in which the corresponding methionine residue in all three human NPY receptors participates in a hydrophobic network with the peptide F. Thus, this case suggests that molecular docking may fail to explicitly annotate some functional residues, although direct experimental validation will be required in the future. It should also be noted that HDOCK does not support C-terminal amidation during docking, which may have affected the quality of the predicted apNPY complex.

(Page. 1)

(Page. 2)

(Page. 3)

(Page. 4)

(Page. 5)

**Figure S9. Complete Sequence alignment of CCK receptors from different species related to Fig. 6.** Amino acid sequences of the transmembrane regions of two *Aplysia* receptors were aligned with those of CCK receptor sequences from representative mollusks, annelids, arthropods, chordates, vertebrates, and nematodes using BioEdit with the MUSCLE multiple alignment algorithm (graphic view). The transmembrane (TM) domains, predicted by DeepTMHMM, are indicated in black lines. The location of conserved motifs of ICL2 (D/ERY) and TM7 (NPXXY) in Class A GPCRs are marked in the alignment. This alignment served as the basis for identifying the topologically equivalent positions discussed in the main text **(Fig. 6)**, including residues corresponding to 5.26, 5.27, and 7.35, which are labeled in this figure. Detailed information about these sequences can be seen in **Table S4**. ap, *Aplysia californica*; Lg, *Lottia gigantea*; Mg, *Magallana gigas*; CtCCKR, *Capitella teleta*; Hr, *Helobdella robusta*; Pd, *Platynereis dumerilii*; Tc, *Tribolium castaneum*; Ds, *Drosophila melanogaster*; Bm, *Bombyx mori*. Ci, *Ciona intestinalis*; Dr, *Danio rerio*; Xl, *Xenopus laevis*; Mm, *Mauremys mutica*; Gg, *Gallus gallus*; Rn, *Rattus norvegicus*; Mu, *Mus musculus*; Hs, *Homo sapiens*; Bt, *Bos taurus*; Ce, C*aenorhabditis elegans*. ECL, Extracellular loop; ICL, Intracellular loop; TM, transmembrane domain.

**Figure S10. Structural basis for the selective sulfation preference of CCK_A_R and the lack of sulfation preference in CCK_B_R.** Each model is shown as an overall receptor structure together with a zoomed-in view of the boxed region, although the overall and enlarged views are not always presented from the same viewing angle. **A**, In cryo-EM-resolved CCK_A_R (PDB: 7EZH) (4), R197^ECL2 (5.26)^ consistently forms a salt bridge with sY^2^, making sulfation a critical interaction determinant in this receptor. **B**, In CCK_B_R (PDB: 7F8V) (5), however, the R at position ECL2 (5.26) is lost. Instead, the adjacent H^ECL2 (5.27)^ forms a strong salt bridge with D^7^ in the DFamide motif, while the ligand sY^2^ no longer interacts directly with the receptor. As a result, sulfation no longer plays a major functional role in CCK_B_R.

**Figure S11. Docking analysis of arthropod *Drosophila* and *Tribolium* SK receptors with their corresponding peptides with sulfated tyrosine (*Drosophila* SK1: FDDYGHMRFamide; *Tribolium* SK2 (5-13): FDDYGHMRFamide).** Each model is shown as an overall receptor structure together with a zoomed-in view of the boxed region, although the overall and enlarged views are not always presented from the same viewing angle. Earlier preliminary docking studies did not yet define an equivalent R^ECL2 (5.26)^-sulfate interaction with comparable clarity (6-8). Re-examination using our docking framework yielded preliminary models in both *Drosophila* (**A**) and *Tribolium* (**B**) in which R^ECL2 (5.26)^ directly contacts the sulfate in the tyrosine residues. Specifically, in the *Drosophila* SK receptor (NP_001097023.1), sY^4^ forms a salt bridge with R264^ECL2 (5.26)^, whereas in the *Tribolium* SK receptor (AGK29938), sY^4^ forms a salt bridge with R204^ECL2 (5.26)^.

**Figure S12. Docking analyses of annelids *Capitella* and *Helobdella CCK* receptors with their corresponding peptides with sulfated tyrosine (*****Capitella* CCK1: pEGAAWDMDYGWGGGRFamide; *Helobdella* CCK: HYDPLGTGGRFamide).** Each model is shown as an overall receptor structure together with a zoomed-in view of the boxed region, although the overall and enlarged views are not always presented from the same viewing angle. Whether annelid CCK-like peptides undergo tyrosine sulfation remains unknown. However, in our comparative models of sulfation recognition **(Fig. 7)**, annelid CCKRs are predicted to lack canonical sulfate recognition because the conserved R^ECL2 (5.26)^ residue is replaced by hydrophobic residues such as I/V. Therefore, if tyrosine sulfation does occur in these peptides as predicted, the sulfate group is not expected to form a productive interaction with ECL2(5.26). Consistent with this prediction, preliminary docking analyses showed that in both annelid systems from *Capitella* (**A**) and *Helobdella* (**B**), neither I/V ^ECL2 (5.26)^ nor the sulfated tyrosine participates in direct binding (*Capitella* CCK receptor: ELT89517.1; *Helobdella* CCK receptor: XP_009030452.1). This recognition pattern resembles the reduced sulfate-recognition mode observed in human CCK_B_R (5), which exhibits low selectivity for sulfated ligands.

**Table S2. Ligand binding potency and expression levels of apCCKR1/apCCKR2 and its mutants.**

| **Receptor** | **BW** | **Mutant** | **Peptides** | **EC_50_** | **pEC_50_** | **Surface expression** |
| --- | --- | --- | --- | --- | --- | --- |
| **apCCKR1** | WT | WT | [sY6,sY8]-CCK1 | 73 | 7.16±0.18 | 100 |
|  |  |  | [sY8]-CCK1 | 159 | 6.80±0.07 |  |
|  |  |  | [sY5]-CCK2 | 880 | 6.06±0.04 |  |
|  | 3.29 | R125A | [sY6,sY8]-CCK1 | 10626 | 4.98±0.06**** | 101.20±3.38 |
|  |  |  | [sY8]-CCK1 | 4951 | 5.33±0.18**** |  |
|  |  |  | [sY5]-CCK2 | 63827 | 4.36±0.49**** |  |
|  | ECL2(5.26) | R200A | [sY6,sY8]-CCK1 | 18600 | 4.73±0.06**** | 102.80±1.60 |
|  |  |  | [sY8]-CCK1 | 10500 | 4.98±0.41**** |  |
|  |  |  | [sY5]-CCK2 | 12600 | 4.90±0.35*** |  |
|  | 6.55 | N344A | [sY6,sY8]-CCK1 | 229 | 6.64±0.11** | 102.83±1.59 |
|  |  |  | [sY8]-CCK1 | 257 | 6.59±0.17** |  |
|  |  |  | [sY5]-CCK2 | 3720 | 5.43±0.30* |  |
|  | ECL3 | R355A | [sY6,sY8]-CCK1 | 71 | 7.18±0.22 ^n.s.^ | 101.57±1.99 |
|  |  |  | [sY8]-CCK1 | 131 | 6.89±0.12 ^n.s.^ |  |
|  |  |  | [sY5]-CCK2 | 832 | 6.08±0.07 ^n.s.^ |  |
|  | 7.35 | K363A | [sY6,sY8]-CCK1 | 3720 | 5.43±0.06**** | 98.64±2.59 |
|  |  |  | [sY8]-CCK1 | 3980 | 5.40±0.15**** |  |
|  |  |  | [sY5]-CCK2 | 11200 | 4.95±0.25*** |  |
|  | 7.43 | Y371A | [sY6,sY8]-CCK1 | 251 | 6.60±0.14** | 102.58±1.95 |
|  |  |  | [sY8]-CCK1 | N.D. | N.D. |  |
|  |  |  | [sY5]-CCK2 | 3090 | 5.51±0.24* |  |
| **apCCKR2** | WT | WT | [sY6,sY8]-CCK1 | 602 | 6.37±0.15 | 100 |
|  |  |  | [sY8]-CCK1 | 42 | 7.42±0.24 |  |
|  |  |  | [sY5]-CCK2 | 87 | 7.07±0.12 |  |
|  | 3.29 | R201A | [sY6,sY8]-CCK1 | 26210 | 4.67±0.33**** | 105.02±8.05 |
|  |  |  | [sY8]-CCK1 | 12900 | 4.89±0.36**** |  |
|  |  |  | [sY5]-CCK2 | 3510 | 5.53±0.32**** |  |
|  | ECL2 | Q263A | [sY6,sY8]-CCK1 | 327 | 6.50±0.18 ^n.s.^ | 107.06±12.43 |
|  |  |  | [sY8]-CCK1 | 143 | 6.89±0.27 ^n.s^ |  |
|  |  |  | [sY5]-CCK2 | 238^.^ | 6.71±0.31 ^n.s^ |  |
|  | ECL2(5.26) | R276A | [sY6,sY8]-CCK1 | 3570 | 5.44±0.10**** | 108.58±6.34 |
|  |  |  | [sY8]-CCK1 | 1927 | 5.72±0.04**** |  |
|  |  |  | [sY5]-CCK2 | N.D. | N.D. |  |
|  | 6.55 | S438A | [sY6,sY8]-CCK1 | 2612 | N.D. | 108.54±5.89 |
|  |  |  | [sY8]-CCK1 | 783 | N.D. |  |
|  |  |  | [sY5]-CCK2 | 562 | 6.25±0.16* |  |
|  | 7.35 | K457A | [sY6,sY8]-CCK1 | 697^.^ | 6.16±0.10 ^n.s^ | 105.70±7.54 |
|  |  |  | [sY8]-CCK1 | 13597 | 4.88±0.10**** |  |
|  |  |  | [sY5]-CCK2 | 708 | 6.15±0.46 ^n.s^ |  |
|  | 7.43 | Y465A | [sY6,sY8]-CCK1 | 12953 | N.D. | 106.45±4.22 |
|  |  |  | [sY8]-CCK1 | 13100 | N.D. |  |
|  |  |  | [sY5]-CCK2 | 2976 | 5.67±0.26*** |  |

This table shows pEC_50_ (M) and EC_50_ (nM) values of three apCCK peptides ([sY6,sY8]-CCK1, [sY8]-CCK1 and [sY5]-CCK2) on apCCKR1 and APCCKR2 and their mutants. The IP1 accumulation assay was performed to evaluate peptide-induced receptor activation. The surface expression (%WT) is detected by performing ELISA (See detailed information in the Method section). Data are presented as means ± SD. of three or more than three independent experiments (n ≥ 3). ND, not detected. All data were analyzed by one-way ANOVA with Tukey’s test. : n.s., not significant; * P < 0.05; ** P < 0.01; *** P < 0.001; **** P < 0.0001. BW, Ballesteros-Weinstein numbering rule; ECL, Extracellular loop; p[EC_50_], -log_10_(EC_50_).

**Table S3. Detailed information of RFamide-like receptors used in Figs. 6-8.**

| **Receptor** | **Subphylum** | **PubMed ID** | **Accession number** |
| --- | --- | --- | --- |
| apCCKR1* | Invertebrate  (Predicted) | PMID: 40700018 | XRO58738.1 |
| apCCKR2* |  | PMID: 40700018 | XRO58739.1 |
| apNPYR |  | / | XP_005104464.1 |
| apLuqinR |  | / | XP_012943577.2 |
| apFRFa-R |  | / | TRINITY_DN21082_c0_g2_i6.p1 |
| apFMRFR |  | / | TRINITY_DN22914_c0_g1_i3.p1 |
| HsCCKAR* | Vertebrate | PMID: 34556862 | NP_000721.1 |
| HsCCKBR* |  | PMID: 39083706 | NP_795344.1 |
| HsNPYR1* |  | PMID: 35507650 | NP_000900.1 |
| HsQRFPR* |  | PMID: 34018631 | NP_937822.2 |
| HsPrRPR* |  | PMID: 36922513 | NP_004239.2 |
| HsNPFFR1* |  | PMID: 32814053 | NP_071429.1 |
| HsNPFFR2* |  | PMID: 12477932 | AAI01637.1 |
| HsKissR* |  | / | ACG60651.1 |

**Table. S4 Detailed information of CCK receptors used in Fig. 6 and Fig. S9.**

| **Species** | **Class** | **PubMed ID** | **Accession number** |
| --- | --- | --- | --- |
| HsCCKAR [*Homo sapiens*] * | Mammals | PMID: 34556862 | NP_000721.1 |
| HsCCKBR [*Homo sapiens*] * |  | PMID: 39083706 | NP_795344.1 |
| MuCCKAR [*Mus musculus*] * |  | PMID: 9099891 | NP_033957.1 |
| MuCCKBR [*Mus musculus*] * |  | PMID: 8978369 | NP_031653.1 |
| RnCCKAR [*Rattus norvegicus*] * |  | PMID: 33422646 | NP_036820.1 |
| RnCCKBR [*Rattus norvegicus*] * |  | PMID: 25601282 | NP_037297.2 |
| BtCCKAR [*Bos taurus*] |  | PMID: 19393038 | NP_001095335.1 |
| BtCCKBR [*Bos taurus*] * |  | PMID: 8851180 | NP_776687.2 |
| GgCCKAR [*Gallus gallus*] * | Birds | PMID: 23443924 | NP_001074970.1 |
| GgCCKBR [*Gallus gallus*] * |  | PMID: 12763638 | NP_001001742.1 |
| XlCCKAR[*Xenopus laevis*] * | Amphibians | PMID: 8700154 | NP_001079277.1 |
| XlCCKBR[*Xenopus laevis*] |  | / | XP_018084499.1 |
| MmCCKAR[*Mauremys mutica*] | Reptiles | / | XP 044875484.1 |
| MmCCKBR[*Mauremys mutica*] |  | / | XP 044845923.1 |
| DrCCKAR [*Danio rerio*] | Fishes | / | XP_697493.2 |
| DrCCKBR [*Danio rerio*] |  | / | CAQ14219.1 |
| CiR1 [*Ciona intestinalis*] * | Chordates | PMID: 14659881 | NP_001027945.2 |
| CiR2 [*Ciona intestinalis*] * |  | PMID: 22289502 | NP_001265909.1 |
| apCCKR1 [*Aplysia californica*] * | Mollusks | PMID: 40700018 | XRO58738.1 |
| apCCKR2 [*Aplysia californica*] * |  | PMID: 40700018 | XRO58739.1 |
| MgCCKR1 [*Magallana gigas*] * |  | PMID: 30401878 | AXF50641.1 |
| MgCCKR2 [*Magallana gigas*] * |  | PMID: 30401878 | AXF50642.1 |
| LgCCK1R [*Lottia gigantea*] |  | PMID: 23254933 | XP_009059889.1 |
| LgCCK2R [*Lottia gigantea*] |  | PMID: 23254933 | XP_009047126.1 |
| LgCCK3R [*Lottia gigantea*] |  | PMID: 23254933 | XP_009047144.1 |
| CtCCKR [*Capitella teleta*] | Annelids | PMID: 23254933 | ELT89517.1 |
| HrCCKR [*Helobdella robusta*] |  | PMID: 23254933 | XP_009030452.1 |
| PdCCKR [*Platynereis dumerilii*] |  | PMID: 26190115 | AKQ63033.1 |
| DsKR1 [*Drosophila melanogaster*] * | Arthropods | PMID: 26109357 | NP_001097021.1 |
| DsKR2 [*Drosophila melanogaster*] * |  | PMID: 26109357 | NP_001097023.1 |
| TcSKR1 [*Tribolium castaneum*] * |  | PMID: 23524001 | AGK29938 |
| TcSKR2 [*Tribolium castaneum*] * |  | / | XP_972750.1 |
| BmSKR [*Bombyx mori*] * |  | PMID: 25261999 | NP_001127744.1 |
| CeCCKR-1 [*Caenorhabditis elegans*] | Nematodes | PMID: 9851916 | NP_491918.3 |
| CeCCKR-2a [*Caenorhabditis elegans*] * |  | PMID: 18339709 | ACA81683.1 |
| CeCCKR-2b [*Caenorhabditis elegans*] * |  | PMID: 18339709 | ACA81684.1 |

**References**

1.	Ding, Y., Zhang, H., Liao, Y.-Y., Chen, L.-N., Ji, S.-Y., Qin, J. *et al.* (2022) Structural insights into human brain–gut peptide cholecystokinin receptors. *Cell Discovery* **8**, 55

2.	Park, C., Kim, J., Ko, S.-B., Choi, Y. K., Jeong, H., Woo, H. *et al.* (2022) Structural basis of neuropeptide Y signaling through Y1 receptor. *Nature Communications* **13**, 853

3.	Iwama, A., Kise, R., Akasaka, H., Sano, F. K., Oshima, H. S., Inoue, A. *et al.* (2024) Structure and dynamics of the pyroglutamylated RF-amide peptide QRFP receptor GPR103. *Nature Communications* **15**, 4769

4.	Liu, Q., Yang, D., Zhuang, Y., Croll, T. I., Cai, X., Dai, A. *et al.* (2021) Ligand recognition and G-protein coupling selectivity of cholecystokinin A receptor. *Nature Chemical Biology* **17**, 1238-1244

5.	Zhang, X., He, C., Wang, M., Zhou, Q., Yang, D., Zhu, Y. *et al.* (2021) Structures of the human cholecystokinin receptors bound to agonists and antagonists. *Nature Chemical Biology* **17**, 1230-1237

6.	Yu, N., Zotti, M. J., Scheys, F., Braz, A. S., Penna, P. H., Nachman, R. J., and Smagghe, G. (2015) Flexibility and extracellular opening determine the interaction between ligands and insect sulfakinin receptors. *Scientific Reports* **5**, 12627

7.	Leander, M., Heimonen, J., Brocke, T., Rasmussen, M., Bass, C., Palmer, G. *et al.* (2016) The 5-amino acid N-terminal extension of non-sulfated drosulfakinin II is a unique target to generate novel agonists. *Peptides* **83**, 49-56

8.	Nichols, R., Bass, C., and Katanski, C. (2022) Structure-activity relationship data and ligand-receptor interactions identify novel agonists consistent with sulfakinin tissue-specific signaling in *Drosophila melanogaster* heart. *Frontiers in Bioscience-Landmark* **27**, 150
